# EPAS1 induction drives myocardial degeneration in desmoplakin-cardiomyopathy

**DOI:** 10.1016/j.isci.2025.111895

**Published:** 2025-01-25

**Authors:** Eirini Kyriakopoulou, Sebastiaan J. van Kampen, Martijn Wehrens, Su Ji Han, Hesther de Ruiter, Jantine Monshouwer-Kloots, Emma Marshall, Andreas Brodehl, Petra van der Kraak, Anneline S.J.M. te Riele, Egidius E.H.L. van Aarnhem, Linda W. van Laake, Hoyee Tsui, Cornelis J. Boogerd, Eva van Rooij

**Affiliations:** 1 Hubrecht Institute, Royal Netherlands Academy of Arts and Sciences (KNAW) and University Medical Center Utrecht, Utrecht, the Netherlands; 2 Erich and Hanna Klessmann Institute, Heart and Diabetes Center NRW, University Hospital of the Ruhr-University Bochum, Bad Oeynhausen, Germany; 3 Department of Pathology, University Medical Center Utrecht, Utrecht, the Netherlands; 4 Division Heart and Lungs, Department of Cardiology, University Medical Center Utrecht, Utrecht, the Netherlands; 5 Netherlands Heart Institute, Utrecht, the Netherlands; 6 Division of Heart and Lungs, Department of Cardiothoracic Surgery, University Medical Center Utrecht, Utrecht, the Netherlands

**Keywords:** Cardiovascular medicine, Cell biology, Transcriptomics

## Abstract

Arrhythmogenic cardiomyopathy (ACM) is frequently attributed to desmosomal mutations, such as those in the desmoplakin (*DSP*) gene. Patients with DSP-cardiomyopathy are predisposed to myocardial degeneration and arrhythmias. Despite advancements, the underlying molecular mechanisms remain incompletely understood, thus limiting therapeutic options. Here, we employed spatial transcriptomics on an explanted heart from a patient with a pathogenic *DSP* variant. Our transcriptional analysis revealed endothelial PAS domain-containing protein 1 (EPAS1) as a potential regulator of mitochondrial homeostasis in stressed cardiomyocytes. Elevated EPAS1 levels were associated with mitochondrial dysfunction and hypoxic stress in both human-relevant *in vitro* ACM models and additional explanted hearts with genetic cardiomyopathy. Collectively, cardiomyocytes bearing pathogenic *DSP* variants exhibit mitochondrial dysfunction, increased apoptosis, and impaired contractility, which are linked to the increased EPAS1 levels. These findings implicate EPAS1 as a key regulator of myocardial degeneration in DSP-cardiomyopathy, which expand to other forms of ACM.

## Highlights

• DSP haploinsufficiency leads to mitochondrial stress in cardiomyocytes
• Mutant cardiomyocytes display elevated ROS levels and mitochondrial dysfunction
• EPAS1 is induced in mutant cardiomyocytes in an ROS-dependent way
• EPAS1 induction drives apoptosis and impaired contractility in mutant cardiomyocytes

## Graphical abstract

**Figure:**
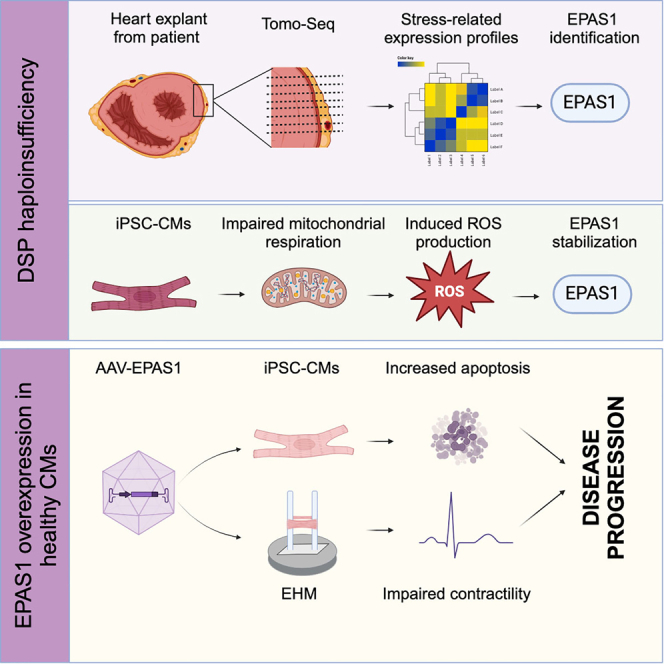

## Introduction

Arrhythmogenic cardiomyopathy (ACM) is a heart condition characterized by severe ventricular arrhythmias and an increased risk of sudden cardiac death, particularly in young athletes. It affects approximately 1 in 2,000 to 1 in 5,000 people worldwide. ACM progresses through three distinct phases: the concealed phase, which is difficult to diagnose due to mild symptoms; the electrical phase, where ventricular arrhythmias develop; and the structural phase, mainly characterized by thinning of the myocardial wall and fibro-fatty tissue infiltration within the cardiac muscle, often leading to heart failure.^1^^,^^2^ Cardiomyocyte loss is considered a common trait of ACM and acts as an arrhythmogenic substrate, consequently increasing the risk of SCD.^3^ Even though the clinical features of ACM have been well-described, diagnosis remains challenging due to high phenotypical variability and incomplete penetrance of the disease.^3^ Moreover, clinical manifestations frequently overlap with dilated cardiomyopathy (DCM) and acute myocardial inflammatory syndromes, thus increasing the risk of misdiagnosis.^4^

Pathogenic variants in desmosomal proteins are the main cause of ACM.^2^^,^^5^ The desmosome is a protein complex residing within the intercalated disc (ID) of cardiomyocytes.^6^^,^^7^ There, it interacts with adherens junctions, gap junctions, and ion channels, together forming a functional unit known as the *area composita*.^8^^,^^9^ The primary role of the desmosome is to physically connect neighboring cardiomyocytes by joining their intermediate filament (IF) networks.^6^ Desmosomal cadherins desmoglein-2 (DSG2) and desmocollin-2 (DSC2), interact with cadherins of opposing cardiomyocytes extracellularly, while their intracellular domains connect with desmoplakin (DSP), plakoglobin (JUP), and plakophilin-2 (PKP2). Besides its stabilizing and adhesive functions, the desmosome plays an essential role in electrical homeostasis in the cardiomyocyte. Multiple studies have linked PKP2 loss to increased arrhythmogenesis by impairing calcium channels, sodium channels and the connexin 43 (CX43) hemichannel function.^10^^,^^11^^,^^12^^,^^13^ Pathogenic variants in the *DSP* gene are also shown to affect CX43 channel functions, contributing to electrical dysfunction in ACM.^14^^,^^15^ Additionally, desmosomes may play a role in the increased inflammatory response seen in ACM patients,^16^ with PKP2 linked to the transcription of genes encoding inflammatory factors and immune system components.^17^ Finally, JUP is known to relocate from IDs to the myocyte nucleus, disrupting transcriptional regulation by the β-catenin/Tcf/Lef complex.^18^ Taken together, the cardiac desmosome constitutes a multifunctional complex that is crucial for maintaining the structural integrity and function of the myocardial wall.

Among desmosomal components, DSP is particularly notable as it is the primary force transducer between desmosomes and IFs. Its critical function is underscored by the fact that variants in *DSP* are directly linked to a wide range of molecular alterations in cardiomyocytes, including ID instability, cardiomyocyte apoptosis, enhanced fibrosis and lipid accumulation.^18^^,^^19^^,^^20^^,^^21^^,^^22^^,^^23^^,^^24^ Recent insights into ACM pathobiology revealed mitochondrial dysfunction as a key aspect of the disease.^25^^,^^26^^,^^27^^,^^28^ Generally, impaired mitochondrial function in cardiac disease is accompanied by elevated levels of reactive oxygen species (ROS), further impacting the integrity of cardiomyocytes.^25^^,^^29^^,^^30^ While these studies have contributed to our understanding of the stress-related mechanisms underlying ACM, the exact molecular players driving these processes have not yet been fully elucidated.

In this study, we applied spatially resolved transcriptomics to an explanted heart from a patient with a heterozygous nonsense variant in *DSP* (c.1705A>T, p.Lys569X). This approach allowed us to identify local activation of a gene expression program associated with cardiomyocyte stress. By focusing on the specific genes expressed in the region containing stressed cardiomyocytes, we identified factors linked to mitochondrial function, indicating mitochondrial stress in these areas. DNA motif analysis within these regions highlighted EPAS1 as a key regulator of this gene profile. Detailed examination of the spatial transcriptomics dataset revealed several potential EPAS1-regulated genes, mostly encoding for proteins directly involved in mitophagy, hypoxic response and free radical scavenging. This suggests a link between mitochondrial dysfunction and EPAS1 induction in diseased cardiomyocytes. Further studies in human induced pluripotent stem cell-derived cardiomyocytes (hiPSC-CMs) harboring a nonsense variant in *DSP* confirmed increased EPAS1 levels in mutant cardiomyocytes, that was accompanied by reduced mitochondrial respiration and heightened ROS production compared to control cardiomyocytes. Overexpression and knockdown experiments further connected the increase in EPAS1 levels with apoptosis, autophagy, and hypoxic stress in human cardiomyocytes. Moreover, these experiments identified EPAS1 as an important factor affecting cardiomyocyte contractility, by reducing force of contraction in engineered human myocardium (EHM). Together, these findings highlight the potential of spatial transcriptomics as a powerful tool for linking molecular processes to local remodeling responses and indicate EPAS1 as a critical molecular driver of cardiomyocyte degeneration in the context of DSP-cardiomyopathy.

## Results

### The pathogenic *DSP*^p.Lys569X^ variant causes cardiac remodeling and arrhythmias

**Figure 1:**
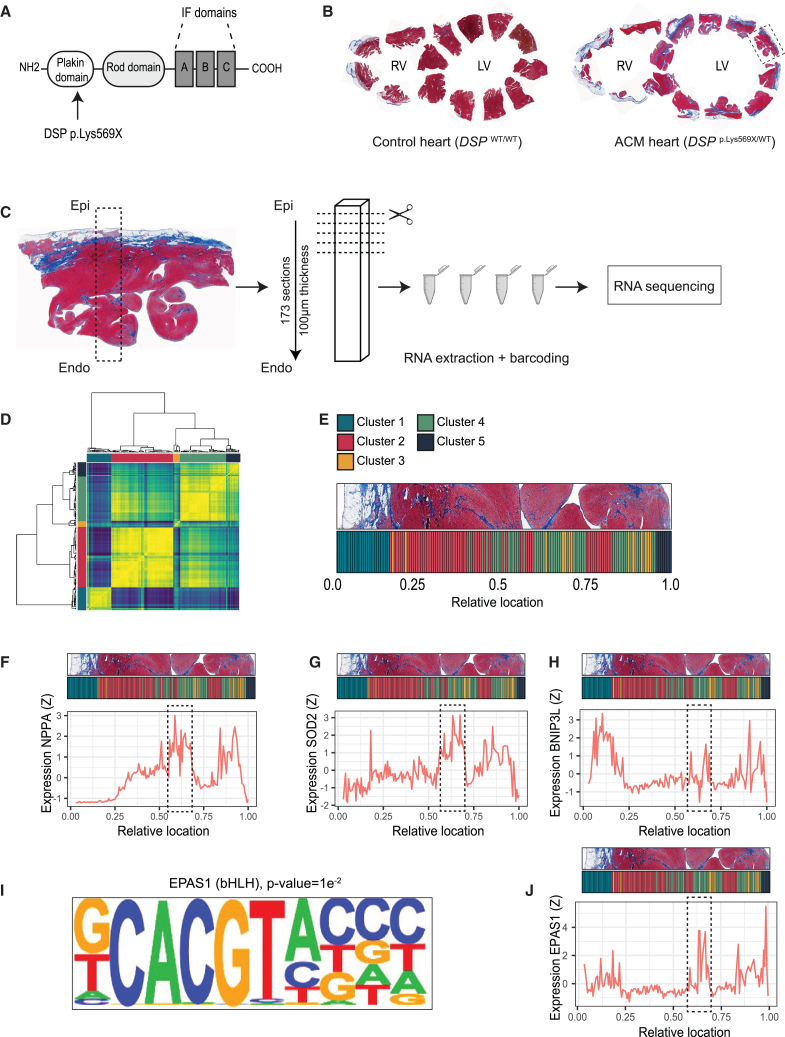
Tomo-seq analysis identified an EPAS1-enriched cardiomyocyte cluster in ACM (A) Schematic representation of desmoplakin (DSP). The arrow indicates the location of the DSP p.Lys569X variant within the plakin domain. IF domain; intermediate filaments domain. (B) Masson’s trichrome staining of non-failing control heart (left) and DSP p.Lys569X ACM patient heart (right). RV: right ventricle; LV: left ventricle. The dashed line marks the LV section utilized in this study. (C) Schematic representation of the Tomo-seq workflow. (D) Pairwise Pearson’s correlation of the sequenced sections. The x axis shows the physical order of the sections from the epi- (bottom) to endocardium (top) and the y axis displays the hierarchically clustering. (E) Cluster identity of sections arranged from the epi- (left) to endocardium (right), aligned to a representative histology image. (F–H) Spatial expression (Z-scores) for cluster 4 enriched genes *NPPA* (F), *SOD2* (G) and *BNIP3L* (H), across all sections. (I) DNA motif analysis on genes enriched in cluster 4 (log2FC > 0.5, adjusted *p* < 0.05) reveals enrichment for EPAS1 binding motif. (J) Spatial expression pattern for *EPAS1* across all sections.

To enhance our understanding of the pathomolecular mechanisms underlying cardiomyopathy development, we conducted an in-depth study of a heart explanted from a patient carrying the heterozygous *DSP* c.1705A>T variant. This genetic variant is predicted to introduce a premature stop codon within the plakin domain at position 569 (*DSP* ^p.Lys569X^; Figure 1A). The patient presented at age 44 with decreased exercise intolerance based on a non-ischemic cardiomyopathy (normal coronary arteries on angiogram). His ECG showed right-sided conduction delay and T-wave inversion in left precordial leads. Echocardiography showed biventricular dilatation and dysfunction. After a sustained VT episode, a secondary prevention ICD was implanted. Soon thereafter, he experienced frank heart failure and received a cardiac transplantation. Gross examination of the explanted heart revealed myocardial degeneration and extensive fibro-fatty infiltration in both ventricular walls, resulting in an almost complete absence of healthy myocardium in the right ventricle (RV) (Figure 1B). Histological analysis of the left ventricle (LV) showed sub-epicardial accumulation of fibro-fatty tissue (Fibro-fatty region) alongside areas mainly composed of cardiomyocytes (Myocardial region) (Figure S1A). This excess adipose tissue was not detected in non-failing control heart tissue (Figure S1A). Targeted sequencing of control and *DSP* ^p.Lys569X/WT^ samples confirmed the presence of the variant (Figure S1B). Immunoblot analysis demonstrated that the genetic variant led to a reduction in total DSP compared to the control heart (Figures S1C and S1D). Moreover, no truncated DSP protein was detected in the diseased heart, suggesting that the mutant transcript is degraded via nonsense-mediated decay (Figure S1C). Together, these findings indicate that the *DSP* p.Lys569X variant leads to DSP haploinsufficiency, thereby causing ACM.

### Identification of a cardiomyocyte population enriched for stress-related and mitochondrial genes

In an effort to obtain gene expression profiles linked to local remodeling responses, we performed spatial transcriptomics (Tomo-seq)^31^ on left ventricular wall tissue of the *DSP* ^p.Lys569X/WT^ heart (Figure 1C). In total, we sequenced 173 sections with a thickness of 100 μm. After quality control (see STAR Methods and Figures S2A–S2D), 153 remaining sections were used for subsequent analyses. Semi-supervised clustering of the samples revealed two transcriptionally distinct areas, likely reflecting the fibro-fatty and myocardial regions (Figure S2D).

To further zoom in on regional differences in gene expression profile, we performed Pearson’s pairwise correlation, which yielded five distinct clusters of sections based on gene expression differences (Figures 1D and 1E). Cluster 1 included sections localizing to the fatty region and was enriched for known markers of adipose tissue such as perilipin-1 (*PLIN1*) (Figure S3A). Furthermore, gene ontology (GO) analysis on genes enriched in this cluster revealed significant representation for GO terms such as “Extracellular exosome” and “Vesicle”, which are in line with the adipose tissue function as a major endocrine system often secreting extracellular vesicles (Figure S3B). For cluster 2, which was enriched for myocardial sections located adjacent to the fatty epicardial region and toward the endocardium, we observed enrichment of genes related to the sarcomere, including titin (*TTN*) (Figures S3C and S3D), suggesting that this cluster corresponds to cardiomyocyte-enriched regions. Cluster 3 included only a few sections, the majority of which localized within the endocardial region. For this cluster, we did not identify any enriched GO terms. Cluster 4 showed strong enrichment for GO terms related to mitochondrial function, which was illustrated by the expression of mitochondrial components such as NADH dehydrogenase [ubiquinone] 1 alpha subcomplex subunit 12 (*NDUFA12*; Figures S3E and S3F). In addition, similar to cluster 2, this cluster also showed an upregulation of *TTN*, indicating that it represents cardiomyocyte-rich regions flanking the cluster 2 sections. Finally, cluster 5 exhibited high expression levels of cardiac fibroblast marker collagen alpha-1(I) chain (*COL1A1;* Figure S3G). This cluster was enriched for GO terms related to collagen and the extracellular matrix, indicative of a cell population enriched with fibroblasts among its sections, which originated from the endocardial side (Figure S3H). Taken together, this analysis identified regions with distinct gene expression profiles, underscoring the ability of spatial transcriptomics to identify local remodeling responses.

To investigate the potential disease-related processes underlying cardiac stress and apoptosis, we searched for regions exhibiting a transcriptional profile indicative of pathological remodeling. In doing so, we identified significant enrichment for genes involved in mitochondrial processes in cluster 4, potentially representing mitochondrial stress or dysfunction. Strikingly, among the top-regulated genes within this cluster, we identified stress-related genes such as Natriuretic Peptide A (*NPPA*), a gene activated in response to a multitude of cardiovascular disorders (Figure 1F).^32^ Other stress-related genes included upregulated during skeletal muscle growth 5 gene (*USMG5*),^33^ proteasome maturation protein (*POMP*),^34^ mitochondrial pyruvate carrier- 1 and 2 (*MPC1*, *MPC2*),^35^ growth arrest and DNA damage-inducible protein (*GADD45A*), alpha-crystallin B chain (*CRYAB*),^36^ GABA type A receptor associated protein like 2 (*GABARAPL2*)^37^ and adrenomedullin (ADM^38^; Table S2). In addition to a plethora of genes encoding mitochondrial subunits (Table S2), this cluster was enriched for superoxide dismutase 2 (*SOD2*; Figure 1G), which is essential for protecting mitochondria from oxidative stress by catalyzing the dismutation of superoxide radicals into oxygen and hydrogen peroxide and BCL2/adenovirus E1B 19 kDa protein-interacting protein 3-like (*BNIP3L*; Figure 1H), which has a major role in mitochondrial autophagy (mitophagy).^39^^,^^40^ To explore potential transcriptional regulators driving the expression of the upregulated genes in cluster 4, we performed DNA motif enrichment analysis.^41^ We found hypoxia-inducible factor 2a (*HIF2a*), also known as *EPAS1*, as a top enriched DNA binding motif (Figure 1I). EPAS1 is a transcription factor that plays a crucial role in the body’s response to low oxygen levels by regulating genes involved in erythropoiesis, angiogenesis, and metabolic adaptation.^42^ The expression pattern of *EPAS1* closely paralleled that of *NPPA*, *BNIP3L*, and *SOD2* (Figure 1J). Together, these data reveal a distinct cardiomyocyte population that is marked by induced *EPAS1* levels and mitochondrial function-related genes, suggesting that cardiomyocytes within this region endure higher levels of mitochondrial stress.

### EPAS1 is induced in cardiac tissue obtained from patients with ventricular dysfunction

**Figure 2:**
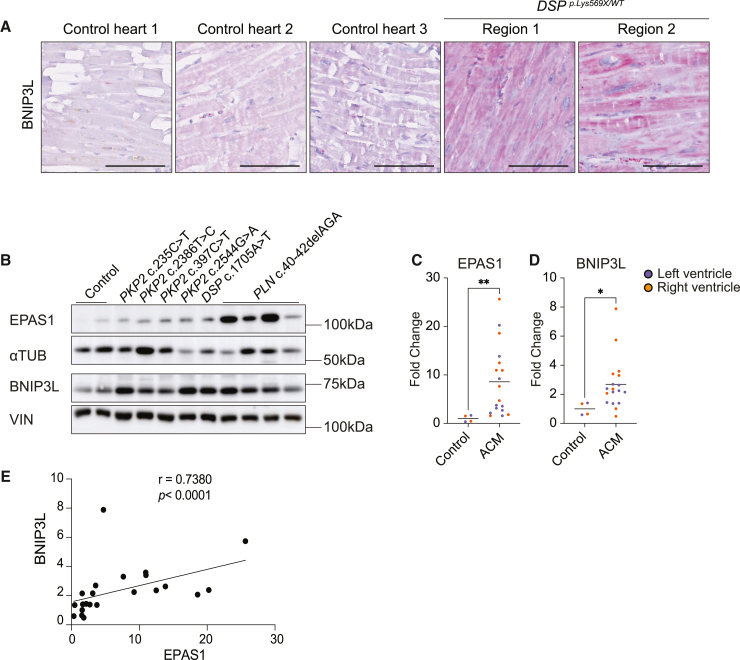
EPAS1 and BNIP3L are induced in human ACM hearts (A) Immunohistochemistry for BNIP3L on left ventricular tissue collected from explanted healthy and DSP p.Lys569X hearts. Scalebar: 100μm. (B) Representative western blot for EPAS1 and BNIP3L on left ventricular tissue obtained from explanted hearts with the indicated mutations. (C) Quantification of EPAS1 protein levels in left- (purple dots) and right- (orange dots) ventricular tissue collected from healthy (two) individuals or patients diagnosed with arrhythmogenic cardiomyopathy (ACM) bearing the indicated mutations. Alpha tubulin (αTUB) was used as a loading control. (D) Quantification of BNIP3L protein levels in left- (purple dots) and right- (orange dots) ventricular tissue collected from healthy (two) individuals or patients diagnosed with arrhythmogenic (ACM) or dilated (DCM) cardiomyopathy bearing the indicated mutations. Vinculin (VIN) was used as a loading control. (E) Spearman’s correlation analysis between EPAS1 and BNIP3L protein levels of the subjects shown in (C-D). A two-tailed Mann-Whitney test was used to assess significance (∗*p* < 0.05, ∗∗*p* < 0.01).

To explore the clinical value of our findings, we performed immunohistochemistry for BNIP3L and SOD2 on the *DSP* p.Lys569X patient’s LV tissue. Compared to control tissue, BNIP3L levels were markedly increased in the myocardial wall of the mutant heart, whereas SOD2 appeared unaffected (Figures 2A and S4A). To investigate whether EPAS1, BNIP3L, and SOD2 induction is a common feature of cardiomyopathy, we also assessed the levels in additional patient hearts with mutations in *PKP2* and phospholamban (*PLN*), associated with ACM and DCM.^43^^,^^44^^,^^45^ Quantification of the protein levels revealed a significant induction for EPAS1 and BNIP3L in left and right ventricular tissue, whereas SOD2 was unaffected (Figures 2B–2D, S4B, and S4C). Correlation analysis of BNIP3L and SOD2 protein expression levels with EPAS1 demonstrated a strong positive correlation with BNIP3L (r = 0.7380; Figure 2E) whilst a weaker correlation with SOD2 was observed (r = 0.0265; Figure S4D). Altogether, EPAS1 and BNIP3L are induced in ACM and DCM hearts, underscoring the relevance of these components in cardiac disease.

### *DSP*^p.Arg1113X/WT^ cardiomyocytes show increased levels of EPAS1 and impaired mitochondrial function

**Figure 3:**
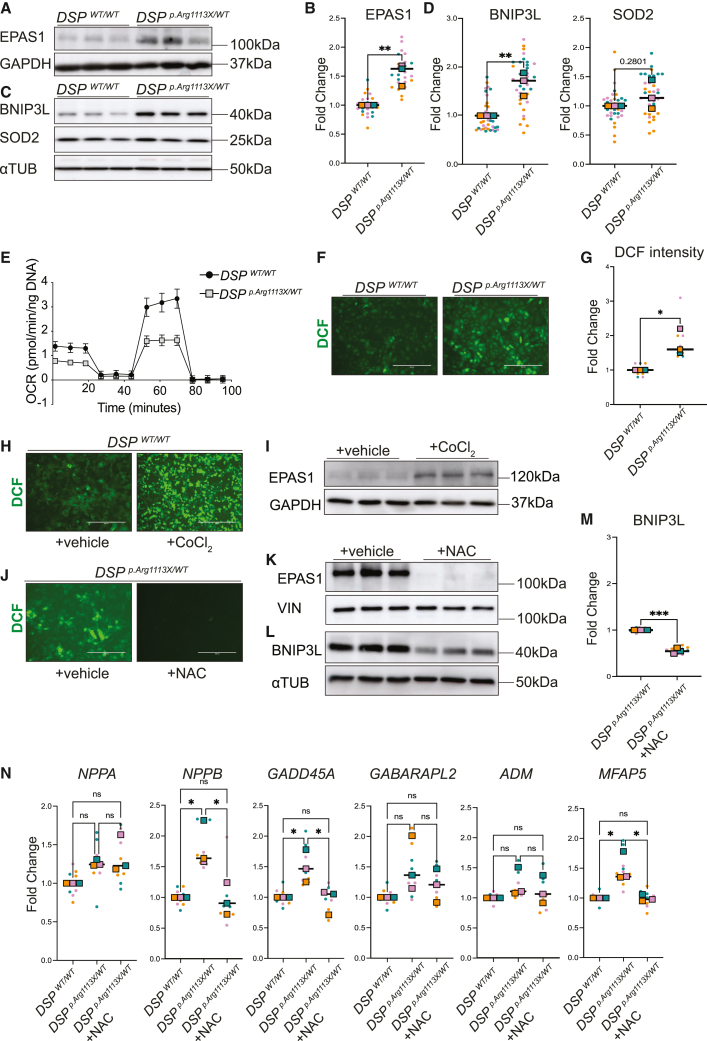
EPAS1 is induced in *DSP*^p.Arg1113X/WT^ hiPSC-CMs (A) Representative western blot for EPAS1 on *DSP*^WT/WT^ and *DSP*^p.Arg1113X/WT^ hiPSC-CMs. (B) Quantification of EPAS1 protein levels in *DSP*^WT/WT^ and *DSP*^p.Arg1113X/WT^ hiPSC-CMs. Glyceraldehyde-3-phosphate dehydrogenase (GAPDH) was used as a loading control. Colored dots represent three independent differentiations (*n* = 12 technical replicates). Data plotted as mean. A two-tailed Student’s *t* test was performed on biological replicates to assess significance (∗∗*p* < 0.01). (C) Representative western blot for BNIP3L and SOD2 on *DSP*^WT/WT^ and *DSP*^p.Arg1113X/WT^ hiPSC-CMs. (D) Quantification of BNIP3L and SOD2 protein levels on *DSP*^WT/WT^ and *DSP*^p.Arg1113X/WT^ hiPSC-CMs. Alpha tubulin (αTUB) was used as a loading control. Colored dots represent different batches of differentiations (*n* = 12 technical replicates, N = 3 biological replicates). Data plotted as mean. A two-tailed Student’s *t* test was performed on biological replicates to assess significance (∗∗*p* < 0.01). (E) Mitochondrial respiration reflected by oxygen consumption rate (OCR) levels in *DSP*^WT/WT^ and *DSP*^p.Arg1113X/WT^ hiPSC-CMs under basal conditions or after addition of oligomycin, FCCP or Rotenone (the graph is derived from one representative batch of differentiations, *n* = 10 technical replicates). (F) Representative images of intracellular ROS levels as indicated by dichlorofluorescein (DCF) signal (in green). Scalebar: 100μm. (G) Fold change of DCF signal intensity in *DSP*^p.Arg1113X/WT^ hiPSC-CMs compared to *DSP*^WT/WT^. Colored dots represent different batches of differentiations (n = 3–4 technical replicates, N = 3 biological replicates). Data plotted as mean. A two-tailed Student’s *t* test was performed on biological replicates to assess significance (∗*p* < 0.05). (H) Representative images of intracellular ROS levels as indicated by DCF signal (in green) in *DSP*^WT/WT^ hiPSC-CMs treated with either cobaltium chloride (CoCl_2_) or vehicle. Scalebar: 100μm. (I) Representative western blot for EPAS1 on *DSP*^WT/WT^ hiPSC-CMs upon treatment with either CoCl_2_ or vehicle. (J) Representative images of intracellular ROS levels as indicated by DCF signal (in green) in *DSP*^p.Arg1113X/WT^ hiPSC-CMs treated with either NAC or vehicle. Scalebar: 100μm. (K) Representative western blot for EPAS1 on *DSP*^p.Arg1113X/WT^ hiPSC-CMs upon treatment with either NAC or vehicle. (L) Representative western blot for BNIP3L on *DSP*^p.Arg1113X/WT^ hiPSC-CMs upon treatment with either NAC or vehicle. (M) Quantification of BNIP3L protein levels on *DSP*^WT/WT^ hiPSC-CMs upon treatment with either NAC or vehicle. Alpha tubulin (αTUB) was used as a loading control. Colored dots represent different batches of differentiations (*n* = 4 technical replicates, N = 3 biological replicates). Data plotted as mean. A two-tailed Student’s *t* test was performed on biological replicates to assess significance (∗∗*p* < 0.01). (N) qRT-PCR for *NPPA, NPPB, GADD45A, GABARAPL2, ADM, MFAP5*. Colored dots represent different batches of differentiations (*n* = 3 technical replicates, N = 3 biological replicates). Data plotted as mean. One-way ANOVA was performed on biological replicates to assess significance (∗*p* < 0.05).

Spatial transcriptomics on the *DSP* ^p.Lys569X/WT^ ACM heart, revealed a cardiomyocyte population likely undergoing mitochondrial stress, indicated by the upregulation of genes involved in mitochondrial function, hypoxic response, apoptosis and autophagy. These findings prompted us to further investigate mitochondrial function in DSP-haploinsufficient *in vitro* models. Utilizing CRISPR/Cas9, we introduced a known ACM-associated nonsense *DSP* mutation in a control hiPSC line in a heterozygous manner (*DSP* c.3337 C>T; DSP p.Arg1113X; NM_004415.4 (DSP); see STAR Methods, Figure S5; Table S3. Cardiomyocytes bearing the *DSP* ^p.Arg1113X/WT^ variant exhibited reduced *DSP* mRNA levels compared to the control line (Figures S6A and S6B), and a similar decline in DSP protein levels (Figures S6C and S6D). First, we assessed the levels of EPAS1 and its potential targets in control and *DSP* ^*p.Arg1113X/WT*^ cardiomyocytes by immunoblotting (Figures 3A–3D) and found that levels of EPAS1 and BNIP3L were induced in response to DSP loss in the mutant hiPSC-CMs, whereas SOD2 levels remained unaffected. In line with this, mitochondrial respiration was reduced in mutant cells compared to the wildtype line, as assessed by oxygen consumption rate measurement under basal conditions and upon treatment with modulators of respiration, including oligomycin, carbonyl cyanide-4 (trifluoromethoxy) phenylhydrazone (FCCP), rotenone, and antimycin (Figure 3E). We then assessed reactive oxygen species (ROS) levels in response to *DSP* haploinsufficiency. Quantification of the fluorescent DCF derivative in hiPSC-CMs confirmed higher ROS levels in the mutant line compared to the wild-type line (Figures 3F and 3G). These findings indicate that the *DSP*^p.Arg1113X/WT^ mutant cardiomyocytes exhibit reduced mitochondrial function, accompanied by an increase in ROS production. In order to assess whether ROS induction is enough to stabilize EPAS1 in the mutant cells, we treated the isogenic control line with cobaltium chloride (CoCl_2_), which is known to cause hypoxia and an increase in ROS levels *in vitro.*^46^ Of note, we observed that treatment with CoCl_2_ resulted in a significant increase in EPAS1 protein levels in the treated *DSP* ^WT/WT^ iPSC-CMs compared to the non-treated control cells (Figures 3H and 3I). In addition to that, suppression of ROS levels in mutant cardiomyocytes with the antioxidant N-acetyl cysteine (NAC) led to complete degradation of EPAS1 protein (Figures 3J and 3K), and to a subsequent downregulation of BNIP3L protein levels (Figure 3L). We further assessed the expression levels of genes enriched within cluster 4 of the Tomo-seq data that are related to cardiac stress, apoptosis, and autophagy. Among these genes, *NPPB*, *GADD45A*, and microfibril-associated protein 5 (*MFAP5*) showed significant upregulation in the mutant line compared to the wild-type line. Of note, treatment with NAC in the mutant cells led to restoration of the expression levels of these genes, preceded by EPAS1 degradation (Figure 3M). In addition, *NPPA*, *GABARAPL2*, and *ADM* showed a trend toward upregulation in the mutant cardiomyocytes but no significant response to NAC-mediated ROS elimination (Figure 3M). The downregulation of EPAS1 and its targets following NAC treatment indicates that EPAS1 induction in mutant cardiomyocytes is directly caused by elevated ROS levels and plays a crucial role in regulating the cardiac stress response. Furthermore, these findings suggest that antioxidants could be employed to preserve viable myocytes in the context of ACM.

### Depletion of DSP in healthy hiPSC-CMs induces the expression of EPAS1 and its target genes

**Figure 4:**
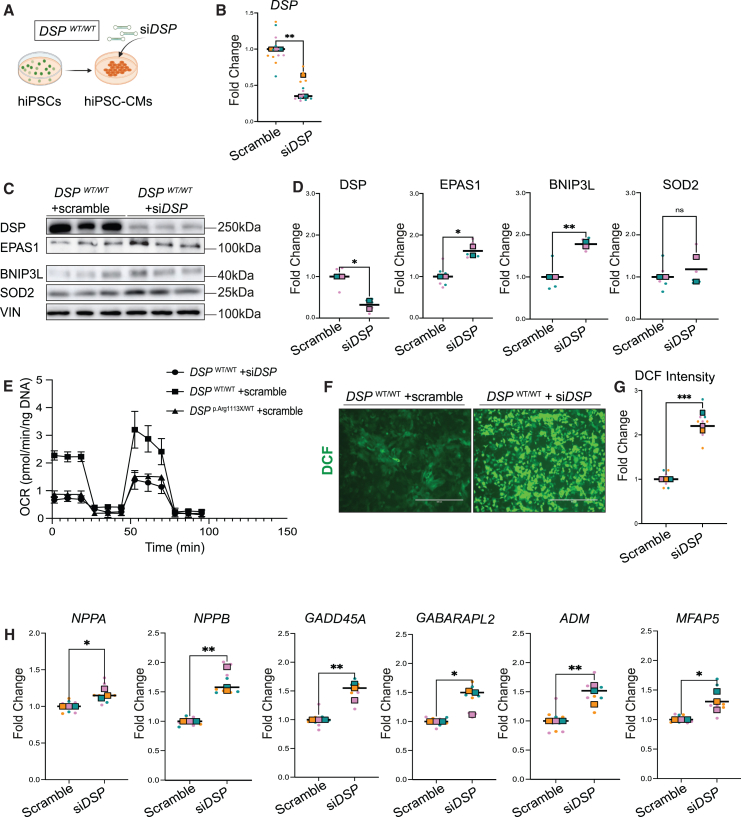
Knockdown of desmoplakin in control hiPSC-CMs induces EPAS1 expression (A) Schematic representation of the workflow followed. (B) Level of expression of *DSP.* Values normalized to the housekeeping gene *GUS*. (C) Representative immunoblot for DSP, EPAS1, BNIP3L, SOD2, and VIN. (D) Quantification of the expression levels of the indicated proteins. Values normalized to VIN. (E) Mitochondrial respiration reflected by oxygen consumption rate (OCR) levels in *DSP*^WT/WT^ hiPSC-CMs treated with either si*DSP* or scramble and *DSP*^p.Arg1113X/WT^ hiPSC-CMs treated with scramble. Measurement was performed under basal conditions or after addition of oligomycin, FCCP or Rotenone (the graph is derived from one representative batch of differentiations, *n* = 10 technical replicates). (F) Representative images of intracellular ROS levels as indicated by dichlorofluorescein (DCF) signal (in green). Scalebar: 100μm. (G) Fold change of DCF signal intensity in *DSP*^WT/WT^ + si*DSP* hiPSC-CMs compared to *DSP*^WT/WT^ + scramble. Colored dots represent different batches of differentiations (*n* = 3–4 technical replicates, N = 3 biological replicates). Data plotted as mean. A two-tailed Student’s *t* test was performed on biological replicates to assess significance (∗*p* < 0.05). (H) RT-qPCR for *NPPA*, *NPPB*, *GADD45A*, *GABARAPL*2, *ADM* and *MFAP5*. Colored dots represent different batches of differentiations (*n* = 3–6 replicates for each batch). Data plotted as mean. A two-tailed unpaired Student’s *t* test or two-tailed Mann-Whitney test was used to assess significance (∗*p* < 0.05, ∗∗*p* < 0.01).

To investigate whether the increase in EPAS1 was a direct consequence of the loss in DSP, we next used a distinct hiPSC-CM model. We treated healthy cardiomyocytes with either control siRNA or siRNA targeting *DSP* for 72 h (Figure 4A). We observed a 70–80% reduction in *DSP* mRNA levels, which corresponded to significant reduction in DSP protein levels (Figures 4B–4D). DSP knockdown led to increased EPAS1 and BNIP3L levels, whereas SOD2 levels remained similar (Figures 4C and 4D). Functionally, DSP knockdown led to mitochondrial dysfunction, as indicated by Seahorse analysis, and significantly increased ROS levels, mirroring the effects observed in *DSP* ^p.Arg1113X/WT^ cells (Figures 4E–4G). Moreover, gene expression analysis indicated significant upregulation of several stress-related genes including *NPPA*, *NPPB*, *GADD45A*, *GABARAPL2*, *ADM*, and *MFAP5*, which aligns with the transcriptional profile characterizing cluster 4 (Figure 4H). These results show that reduced DSP levels, simulating haploinsufficiency, lead to mitochondrial stress and activate EPAS1 along with its downstream target genes.

### EPAS1 induction in hiPSC-CMs associates with increased apoptosis and impaired contractility

**Figure 5:**
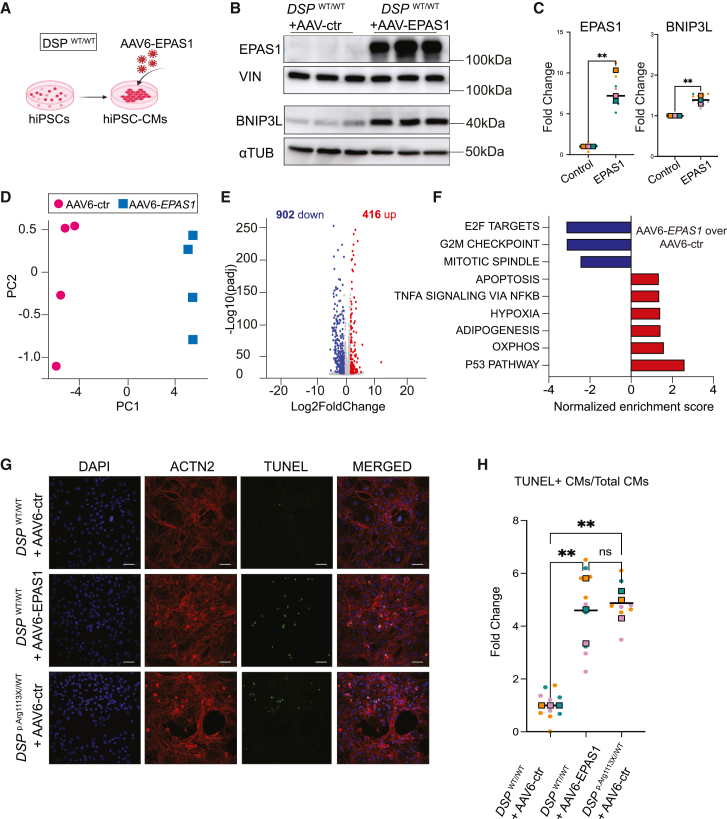
EPAS1 overexpression induces apoptosis in hiPSC-CMs (A) Transduction of *DSP*^WT/WT^ hiPSC-CMs with AAV6-EPAS1 or control virus (AAV6-ctr). (B) Representative western blot of EPAS1 (top) and BNIP3L (bottom) on *DSP*^WT/WT^ hiPSC-CMs upon transduction with either AAV6-ctr or AAV6-EPAS1. (C) Quantification of EPAS1 (left) and BNIP3L (right) protein levels on *DSP*^WT/WT^ hiPSC-CMs upon transduction with either AAV6-ctr or AAV6-EPAS1. Vinculin (VIN) and alpha tubulin (αTUB) were used as loading controls for EPAS1 and BNIP3L quantification, respectively. Colored dots represent different batches of differentiations (*n* = 3 technical replicates, N = 3 biological replicates). Data plotted as mean. A two-tailed Student’s *t* test was performed on biological replicates to assess significance (∗∗*p* < 0.01). (D) Principal component analysis (PCA) of RNA-sequencing results. (E) Volcano plot of Log2 fold change versus Log10 adjusted *p* showing regulated genes after EPAS1 overexpression versus control. (F) Gene set enrichment analysis (GSEA) revealing pathways enriched and suppressed after EPAS1 overexpression. (G) Representative immunostaining for apoptosis (TUNEL; green), counterstained with ACTN2 (red) and DAPI (blue). Scalebar: 10 μm. (H) Quantification of the TUNEL-positive cardiomyocytes from (G). Data are presented as a fold change compared to the control condition (*DSP*^WT/WT^ + AAV6-ctr). Colored dots represent different batches of differentiations (*n* = 3–4 technical replicates, N = 3 biological replicates). Data plotted as mean. One-way ANOVA was performed on biological replicates to assess significance (∗*p* < 0.05).

To investigate the role of EPAS1 induction in cardiomyocytes, we overexpressed *EPAS1* in wild-type hiPSC-CMs using adeno-associated virus serotype 6 (AAV6)-mediated delivery (Figures 5A–5C). Consistent with our previous data, EPAS1 overexpression lead to an increase in BNIP3L protein levels, confirming a regulatory relationship between the two factors (Figures 5B and 5C). Subsequently, we performed RNA sequencing (RNA-Seq) to delineate gene programs regulated by the increase in EPAS1 levels. EPAS1 overexpression led to 902 downregulated genes (log2FC < −0.5, adjusted *p* < 0.05) and 416 upregulated genes (log2FC > 0.5, adjusted *p* < 0.05) compared to control cardiomyocytes (Figures 5D and 5E). Notably, there was a significant enrichment of upregulated genes related to hypoxia and oxidative phosphorylation, corroborating Tomo-seq data (Figure 5F). Overexpression of *EPAS1* was also associated with significant upregulation of genes related to apoptosis and the p53 pathway, a stress response pathway activated by post-translational modifications to the p53 protein, which initiates cell-cycle arrest, cellular senescence, or apoptosis^47^ (Figure 5F). These data show that EPAS1 induces cardiomyocyte apoptosis to a comparable extent as observed in mutant hiPSC-CMs. Next, we sought to validate the results from high-throughput RNA sequencing in our *DSP* mutant and wild-type cardiomyocyte lines. Using terminal deoxynucleotidyl transferase dUTP nick end labeling (TUNEL) to identify double-strand DNA breaks characteristic of apoptotic DNA fragmentation, we demonstrated that *EPAS1* overexpression significantly increased the fraction of apoptotic cardiomyocytes (Figure 5G). This finding confirmed our hypothesis that EPAS1 induction can trigger apoptosis in these cells. Strikingly, *DSP*^p.Arg1113X/WT^ mutant cardiomyocytes treated with the AAV6-control virus exhibited a markedly higher proportion of TUNEL-positive cells compared to wildtype cells receiving the same treatment, indicating an elevated baseline level of apoptosis in the mutant cells (Figures 5G and 5H).

**Figure 6:**
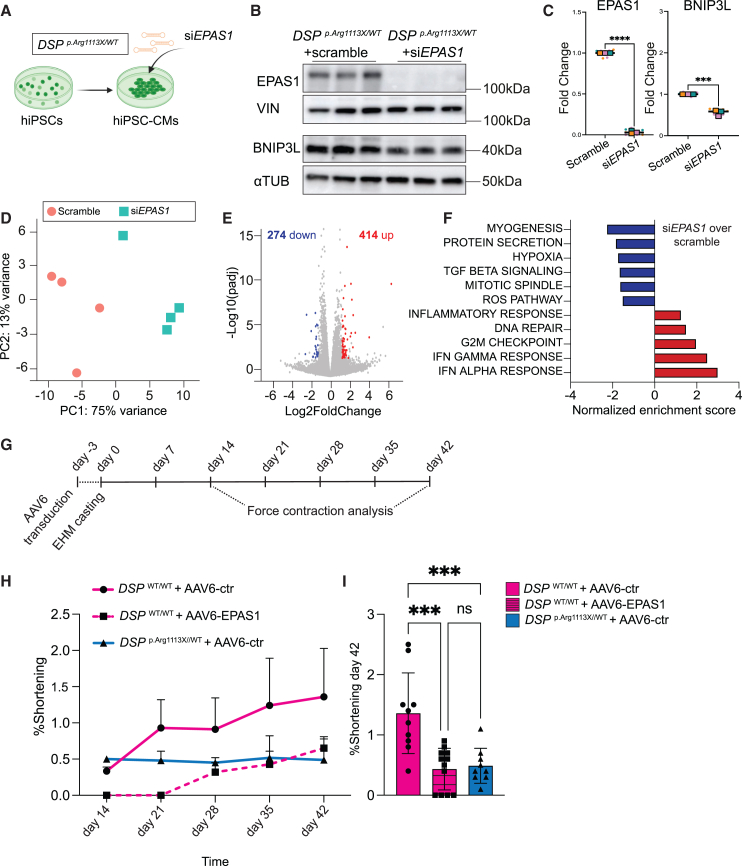
EPAS1 overexpression impairs contractility in engineered human myocardium (A) Small interfering RNA- (siRNA-) mediated knock-down of EPAS1 on *DSP*^p.Arg1113X/WT^ hiPSC-CMs. (B) Representative western blot of EPAS1 (top) and BNIP3L (bottom) on *DSP*^p.Arg1113X/WT^ hiPSC-CMs upon transfection with either si*EPAS1* or scramble (control). (C) Quantification of EPAS1 (left) and BNIP3L (right) protein levels on *DSP*^p.Arg1113X/WT^ hiPSC-CMs upon transfection with either si*EPAS1* or scramble. Vinculin (VIN) and alpha tubulin (αTUB) were used as loading controls for EPAS1 and BNIP3L quantification respectively. Colored dots represent different batches of differentiations (*n* = 3 technical replicates, N = 3 biological replicates). Data plotted as mean. A two-tailed Student’s *t* test was performed on biological replicates to assess significance (∗∗∗∗*p* < 0.0001). (D) PCA of RNA-sequencing results. (E) Volcano plot of Log2 fold change versus Log10 adjusted *p* showing regulated genes after EPAS1 knock-down versus control. (F) GSEA revealing pathways enriched and suppressed after EPAS1 knock-down. (G) Timeline overview for the casting and contraction analysis of engineered heart muscle (EHM). (H) Force of contraction measurements over time. The graph shows mean with standard error of the mean. (I) Force of contraction measurements of AAV6-ctr- or AAV6-EPAS1- treated EHM at 6 weeks after casting. Data plotted as mean, n(*DSP*^WT/WT^ + AAV6-ctr) = 10 tissues, n(*DSP*^WT/WT^ + AAV6-EPAS1) = 12 tissues, n(*DSP*^p.Arg1113X/WT^ + AAV6-ctr) = 9 tissues. One-way ANOVA was performed to assess significance (∗∗∗*p* < 0.001).

To investigate the contribution of EPAS1 induction to cardiomyocyte apoptosis in DSP mutant hiPSC-CMs, we performed *EPAS1* silencing by transfecting *DSP* ^p.Arg1113X/WT^ hiPSC-CMs with a siRNA targeting *EPAS1* (si*EPAS1*) or a control siRNA (scramble; Figures 6A–6C). *EPAS1* knockdown resulted in the reduction of BNIP3L protein levels and significant gene expression changes, with 414 genes upregulated (log2FC > 0.5, adjusted *p* < 0.05) and 274 genes downregulated (log2FC < −0.5, adjusted *p* < 0.05) compared to control cardiomyocytes (Figures 6D and 6E). Notably, among the downregulated genes, there was a marked decrease in the expression of genes related to myogenesis and contractile function (Figure 6F). Furthermore, EPAS1 knockdown led to downregulation of processes such as hypoxia, ROS pathway, and protein secretion, consistent with observations from the *EPAS1* overexpression experiment (Figure 6F). These results further strengthen the association between EPAS1 and stress-related processes such as apoptosis and hypoxic response, along with a reduction in cell cycle genes and myogenesis.

Given the observed link between EPAS1 and myogenesis, we sought to further investigate the impact of EPAS1 induction on contractile function. To this end, we transduced wildtype and *DSP* mutant hiPSC-CMs with either AAV6-EPAS1 or AAV6-control viruses. Three days post-transduction, the cardiomyocytes were reconstituted into engineered human myocardium (EHM), comprising 70% hiPSC-CMs and 30% human foreskin fibroblasts (HFF).^48^ We monitored the contractile function of the tissues through sequential video-recordings of their beating at various time points over six weeks (Figure 6G). Analysis of these recordings revealed that AAV6-EPAS1-treated EHM exhibited a sharp decrease in the force of contraction, indicated by a significant drop in percentage shortening throughout the experiment (Figures 6H and 6I). Similarly, EHM composed of *DSP* mutant hiPSC-CMs combined with HFF showed a significant decrease in force of contraction compared to wild-type tissues (Figures 6H and 6I). These results indicate that EPAS1 induction is sufficient to impair the contractile function of cardiomyocytes.

Together, these findings suggest that EPAS1 induction in cardiomyocytes can activate a gene network triggering apoptosis, hypoxic stress, and autophagy, subsequently leading to myocyte degeneration and impaired contractile function. This is particularly important as it suggests that EPAS1 may play a crucial role in driving cardiomyocyte loss in the context of ACM.

## Discussion

ACM is a pleiotropic disease characterized by electrical and structural dysfunction of cardiomyocytes, that is frequently due to pathogenic variants in desmosomal genes.^43^ Yet, the pathways affected by these variants are still not fully understood, hindering the development of curative treatments.^49^ In this work, we utilize explanted hearts with *DSP*, *PKP2* and *PLN* variants and independent patient-specific *in vitro* models to decipher pathomolecular mechanisms driving cardiomyocyte loss in the context of the disease.

Using spatial transcriptomics on the left ventricular free wall of a heart with a heterozygous *DSP* ^*p.Lys569X/WT*^ mutation, we identified five clusters with distinct gene expression profiles. Subsequent analysis revealed a myocardial cluster enriched for the stress marker *NPPA* and several genes associated with mitochondrial function, autophagy, and apoptosis. This cluster was adjacent to cluster 2, which was highly enriched for cardiac genes such as cardiac troponin (*TNNT2*), *TTN*, and α actinin 2 (*ACTN2*), indicating a separate population of cardiomyocytes undergoing higher levels of mitochondrial stress and potential degeneration. Follow-up analysis allowed us to identify elevated levels of EPAS1, a protein important in the adaptive cellular response to stress.^50^ Under conditions of homeostasis, EPAS1 is rapidly hydroxylated by prolyl hydroxylase domain-containing (PHD) enzymes and targeted for degradation; however, hypoxia results in reduced levels of PHD proteins, thereby increasing the pool of stabilized EPAS1. Consequently, EPAS1 translocates to the nucleus where it binds to hypoxia responsive elements that are present in genes important for metabolic homeostasis, extracellular signaling and apoptosis.^51^^,^^52^ Importantly, increased ROS levels have previously been linked to stabilization of EPAS1.^53^ This is of interest as increased levels of ROS are regularly identified in cardiac disease, including heart failure, cardiac hypertrophy, diabetic cardiomyopathy, cardiac arrhythmias, and more recently also in ACM.^17^^,^^29^^,^^30^ In support of this, we observed induced levels of EPAS1 in cardiomyocytes carrying a heterozygous nonsense variant in *DSP*, which was paralleled by impaired mitochondrial respiration and heightened ROS production. Notably, an increase in EPAS1 levels and its downstream targets was also identified upon DSP knockdown on healthy cardiomyocytes. Together, these data indicate that loss of DSP can cause mitochondrial damage, subsequently leading to EPAS1 induction.

Our data also show that the induction of EPAS1 in mutant cardiomyocytes led to the subsequent upregulation of genes involved in mitochondrial function. BNIP3L is a mitochondrial protein residing within the outer membrane and has been linked to mitophagy, a conserved process that governs mitochondrial integrity.^54^^,^^55^^,^^56^ In addition, several studies revealed a role for BNIP3L in apoptosis and necrosis through its interaction with apoptosis regulator BAX (BAX) and bcl-2 homologous antagonist/killer (BAK).^57^^,^^58^^,^^59^ In hypertrophic cardiomyopathy, hypoxia-independent induction of *BNIP3L* promotes cardiomyocyte death and drives the transition toward acquired DCM.^60^^,^^61^ Interestingly, loss of cardiomyocytes via apoptotic and necrotic events is also considered a hallmark of ACM pathogenesis.^62^^,^^63^ Our Tomo-seq data reveal that BNIP3L is highly expressed not only in cluster 4 but also in cluster 1. This latter cluster, situated on the epicardial side of the heart, predominantly consists of adipocytes and fibroblasts. Given that the current manuscript focuses on cardiomyocyte function in ACM, we have primarily investigated the role of BNIP3L in the context of cardiomyocyte stress. However, the potential involvement of BNIP3L in other cell types is indeed significant and warrants further exploration in future studies.

Similarly, the manganese superoxide dismutase protein SOD2 localizes at mitochondria where it regulates ROS homeostasis via its ability to scavenge free radicals.^64^ Intriguingly, *Epas1*^−/−^ mice display reduced *Sod2* expression levels and show a strikingly similar mitochondrial phenotype as observed for *Sod2*^−/−^ mice, suggesting a direct link between EPAS1 and SOD2.^39^^,^^65^ Furthermore, *Sod2*^−/−^mice show reduced cardiac activity of fundamental mitochondrial enzymes and die from a DCM-like phenotype.^36^ Conversely, cardiomyocyte-specific overexpression of *Sod2* provided protection against oxidative stress and improved mitochondrial respiration in a mouse model of diabetes.^66^^,^^67^ Our data demonstrate that EPAS1 is a key regulator of BNIP3L highlighting its pivotal role in mitochondrial function and cardiomyocyte survival. However, SOD2 seems to not follow the same trends in our systems; in cardiomyocytes, several compensatory mechanisms can limit EPAS1-dependent induction of SOD2. HIF-1α often dominates over EPAS1 under hypoxic conditions, competing for transcriptional control and reducing SOD2 expression.^68^ Additionally, robust antioxidant systems, such as catalase and glutathione peroxidase, along with the Nrf2 pathway, can mitigate oxidative stress without requiring SOD2 upregulation.^69^ Cardiomyocytes also undergo metabolic reprogramming through AMPK and mTOR signaling, shifting energy production away from mitochondria, thereby reducing ROS generation and the need for SOD2.^70^ Moreover, mechanisms like autophagy remove damaged mitochondria, reducing ROS directly, while oncogenic factors like c-Myc and epigenetic modifications can suppress SOD2 transcription.^71^ Last, changes in calcium signaling and mitochondrial biogenesis can also help maintain redox balance without relying on SOD2 induction.^72^ In addition to BNIP3L upregulation in the *DSP* mutant cells, EPAS1 induction also led to the upregulation of several other genes including *NPPA*, *NPPB*, *GADD45A*, *GABARAPL2*, *ADM*, and *MFAP5*, all associated with cardiac stress, apoptosis, and autophagy in cardiomyocytes. Notably, *NPPB* and *GADD45A* showed significant restoration to physiological levels upon EPAS1 downregulation, either through ROS elimination or siRNA-mediated knockdown in the DSP mutant cells, while the other genes exhibited a decreasing trend. These findings are crucial as they suggest an intrinsic mechanism within *DSP*-mutant cardiomyocytes that induces EPAS1, subsequently leading to an increased cardiomyocyte stress response and activation of a degeneration gene program. This gene program is reversed upon EPAS1 degradation in these cardiomyocytes.

Whether EPAS1 induction is an adaptive or a deleterious response to cardiomyocyte stress so far remained controversial. In ischemic heart disease, acute HIF1α activation can be beneficial as a response to acute cardiac stress. HIF activates genes that sustain energy and survival in low-oxygen environments, suggesting that HIF activation is beneficial in acute ischemia, despite being harmful in chronic activation.^73^^,^^74^ Moreover, a recent study from Sadek et al. revealed that EPAS1 overexpression in cardiomyocytes elicits cardiac regeneration through prevention of DNA damage, thus improving systolic function after myocardial infarction in adult mice.^75^ However, our research suggests that the prolonged induction of EPAS1, as a consequence of elevated ROS levels, may additionally induce pathological characteristics of ACM such as autophagy, apoptosis and impaired contractility.^76^^,^^77^^,^^78^ Similarly, a study from Moslehi et al., demonstrated that the HIF pathway plays a critical role in the development of cardiomyopathy, particularly under conditions of long-term inactivation of PHD2 in cardiomyocytes, subsequently leading to long-term stabilization of EPAS1 in these cells.^79^ Long-term EPAS1 activation resulted in mitochondrial loss due to decreased biogenesis, increased autophagy, DCM and premature mortality.^79^ Supporting this idea, a study by May and colleagues used a reversible transgene to induce myocardial ischemia in mice without causing overt infarction.^80^ They observed that ischemia caused EPAS1 activation (indicating impaired PHD function), metabolic reprogramming, mitochondrial autophagy, and systolic dysfunction, all of which were reversed upon removal of the transgene.^80^ Collectively, these results support that EPAS1 plays a causal role in the development of cardiomyopathy.

Together, it seems that the duration of EPAS1 activation is crucial. Strict control of EPAS1 activity via a temporal burst of EPAS1 downstream signaling may be beneficial to cardiac function by allowing cardiomyocytes to respond to transient hypoxia. With the emerging view of the importance of HIF1α and HIF2α in disease, there is a largely unmet need for specific HIF inhibitors. Given that antioxidants such as vitamins C and E, resveratrol, and statins can mitigate oxidative stress and have shown potential in treating various cardiac arrhythmias, they may also offer beneficial effects for patients with DSP-cardiomyopathy.^81^ While combined inhibition of HIFα isoforms will be appropriate in certain disease situations, HIF1α- or HIF2α-specific therapies may be preferable in other scenarios. Further research is required to decipher whether drug-mediated control of EPAS1 induction would be sufficient to impede or prevent disease development in DSP-cardiomyopathy and in ACM associated with genetic defects in other desmosomal genes.

In conclusion, our data indicate EPAS1 to function as a crucial regulator of cardiomyocyte dysfunction in *DSP*-related disease. These findings contribute to our understanding of DSP-related cardiomyopathy and ACM pathogenesis, and may facilitate the development of effective therapeutic strategies.

### Limitations of the study

This study comes with several limitations. Our *in vitro* models are based on hiPSCs which, despite being valuable tools for disease modeling, exhibit an immature phenotype. Consequently, the processes observed in these models may differ significantly from those occurring in the hearts of end-stage patients, potentially leading to inconsistencies between the two models. Additionally, discrepancies between our *ex vivo* and *in vitro* models may arise due to the lack of multicellular communication in our *in vitro* system. Although Tomo-seq provides spatial resolution, it does not yield data from specific cell types of interest. Instead, it aggregates information from various cardiac populations within each section. While our *in vitro* data confirms that EPAS1 induction is relevant for cardiomyocytes, co-culturing with other cardiac cell types, such as fibroblasts and endothelial cells, could provide further insights into the underlying processes. Additionally, in our *in vitro* systems, we observed discrepancies in the expression of several target genes identified through Tomo-seq data analysis (Figure 3N). We hypothesize that these discrepancies arise because iPSC-CMs, being an immature model, do not fully recapitulate the complex physiology of an end-stage patient’s heart. While iPSC-CMs are valuable for studying genetic cardiomyopathy, they lack the structural, metabolic, and electrophysiological maturity of adult cardiomyocytes. In contrast, the end-stage heart has undergone extensive remodeling, fibrosis, and other pathological changes that significantly alter gene expression patterns. Thus, the absence of differential expression in iPSC-CMs likely reflects their inability to fully mimic the advanced disease state seen in patients, which may explain the observed differences in gene expression between the two models. Moreover, this study employs two *in vitro* models of DSP haploinsufficiency to corroborate experimental results: a *DSP* mutant line harboring a patient-specific mutation and a healthy line with *DSP* knockdown. Using an additional line with a different patient-specific *DSP* variant or other desmosomal variant would strengthen the findings and allow for broader extrapolation to more ACM variants. Lastly, while this paper highlights EPAS1 involvement in DSP-cardiomyopathy progression, it does not address the precise mechanism by which a loss in DSP protein levels induces EPAS1 expression. Further research is necessary to elucidate the triggers of mitochondrial dysfunction and subsequent EPAS1 stabilization in this context.

## Resource availability

### Lead contact

Further information and requests for resources and reagents should be directed to and will be fulfilled by the lead contact, Eva van Rooij (e.vanrooij@hubrecht.eu).

### Materials availability

This study did not generate new unique reagents.

### Data and code availability

• RNA-sequencing and Tomo-sequencing data have been deposited at Gene Expression Omnibus and are publicly available as of the date of publication. Accession numbers are listed in the key resources table. Microscopy data reported in this paper will be shared by the lead contact upon request.
• Analysis scripts have been deposited at GitHub and are publicly available as of the date of publication. DOIs are listed in the key resources table.
• Any additional information required to reanalyze the data reported in this paper is available from the lead contact upon request.

## Acknowledgments

This project is supported by the 10.13039/100018890Dutch Cardiovascular Alliance with support of the Dutch Heart Foundation - DCVA 2017-18 ARENA-PRIME (E.v.R), the Vici grant from the 10.13039/501100003246Dutch Research Council - project 09150181910020 (E.v.R) and by the Fondation Leducq Transatlantic Network of Excellence - 17CVD02 (E.v.R). A.S.J.M.t.R. is supported by a 10.13039/501100001826ZonMw grant (Off Road 2021). We thank Farhad A. Moqadam, Ana Rita Leitoguinho, Sjoerd J. Klaasen, and Geert J.P.L. Kops for help with single guide RNA (sgRNA) selection and the karyo-sequencing procedure.

## Author contributions

Conceptualization: E.v.R., E.K., and S.J.v.K. Data collection: E.K., S.J.v.K., S.H., H.d.R., J.M.-K., A.S.J.M.t.R., and E.M. Resources: M.W., A.B., P.v.d.K., N.P.v.d.K., and L.W.v.L. Data analysis: E.K., S.J.v.K., H.T., and C.J.B. Funding acquisition: E.v.R. Writing—original draft: E.K., S.J.v.K., and E.v.R. Writing—review and editing: all authors.

## Declaration of interests

E.v.R. is a consultant for Tenaya Therapeutics and Novo Nordisk and is Chief Scientific Officer of Phlox Therapeutics. A.S.J.M.t.R. is a consultant for Tenaya Therapeutics, Rocket Pharmaceuticals, BioMarin, and Lexio. All other authors declare that they have no competing interests.

## STAR★Methods

### Key resources table

**Table:** 

REAGENT or RESOURCE	SOURCE	IDENTIFIER
**Antibodies**		

Alpha-tubulin (αTUB)	Sigma-Aldrich	AB_477593
BCL2/Adenovirus E1B 19 kDa protein-interacting protein 3-like (BNIP3L)	Cell Signaling Technology	AB_2688036
Endothelial PAS domain-containing protein 1 (EPAS1)	Novus Bio	AB_10002593
Superoxide dismutase [Mn], mitochondrial (SOD2)	Abcam	AB_300434
Vinculin (VIN)	Santa Cruz	AB_628438
Glyceraldehyde-3-phosphate dehydrogenase (GUS)	Sigma-Aldrich	AB_2107445

**Biological samples**		

Human cardiac tissue with ACM/DCM: DSP c.1705A>T (p.K569∗)	This paper	N/A
Human cardiac tissue with ACM: PKP2 c.235C>T (p.Arg79X)	This paper	N/A
Human cardiac with ACM: PKP2 c. 2368T>C (p. Cys796Arg), NM_004572.4	This paper	N/A
Human cardiac with ACM: PKP2 c.397C>T (p.Gln133X), NM_001005242.2	This paper	N/A
Human cardiac with ACM: PKP2 c. 2544G>A (p.Trp848X), NM_004572.4	This paper	N/A
Human cardiac with ACM: PLN c.40-42delAGA (p.Arg14del)	This paper	N/A
Healthy human cardiac tissue (control 1)	This paper	N/A
Healthy human cardiac tissue (control 2)	This paper	N/A

**Chemicals, peptides and recombinant proteins**		

2',7'-dichlorodihydrofluorescein diacetate (H2DCFDA)	Invitrogen	D399
N-acetyl cysteine (NAC)	Sigma Aldrich	1124220025
Thiazovivin (TZV)	Calbiochem	20220-10mg
GSK-3 Inhibitor XVI (CHIR99021)	Calbiochem	361559
Wnt Antagonist II (IWP-2)	Calbiochem	681671

**Critical commercial assays**		

*In Situ* Cell Death Detection Kit, Fluorescein	Roche	11684795910
iScript Synthesis kit	BioRad	#1708891
GoTaq® Green Master Mix	Promega	#9PIM712
Lipofectamine™ RNAiMAX Transfection Reagent	ThermoFisher Scientific	13778075

**Deposited data**		

Tomo-Seq data	This paper	To be provided
Bulk RNA-seq data	This paper	To be provided

**Experimental models: Cell lines**		

*DSP*^WT/WT^	ATCC	ATCC-BYS0112
*DSP*^p.Arg1113X/WT^	N/A	N/A
Human Foreskin Fibroblasts	ATCC	SCRC-1041

**Oligonucleotides**		

Single guide RNA sequences, please see Table S3	Integrated DNA technologies	N/A
Genotyping primers, please see Table S4	Integrated DNA technologies	N/A
Single-stranded templates used for targeting, please see Table S5	Integrated DNA technologies	N/A
Primers for amplification of off-target loci, please see Table S6	Integrated DNA technologies	N/A
Primers for RT-PCR, please see Table S7	Integrated DNA technologies	N/A
siRNA oligo duplexes targeting *DSP*	OriGene, Rockville, MD	CAT#: SR301285
siRNA oligo duplexes targeting *EPAS1*	OriGene, Rockville, MD	CAT#: SR301415

**Software and algorithms**		

GraphPad PRISM v9	GraphPad Prism Inc	https://www.graphpad.com
ImageJ v1.51	NIH	https://imagej.net/ij/
NDP.view2 Image viewing software	Hamamatsu	www.hamamatsu.com/all/en/U12388-01.html
Adobe inDesign CC 2019	Adobe Systems Incorporated	https://www.adobe.com/
BioRender	BioRender	https://www.biorender.com/
R Studio v.4.1.0	RStudio	https://cran.r-project.org/
Custom Scripts-Tomo-Seq	This paper	https://github.com/vanrooij-lab/tomoseq_mw
Bulk RNA-seq: DESeq2	Love et al., 2014^82^	https://bioconductor.org/packages/release/bioc/html/DESeq2.html
GSEABase (v. 1.54.0)	Morgan et al., 2021	https://bioconductor.org/packages/release/bioc/html/GSEABase.html
STAR (v. 2.7.8a)	(Dobin et al., 2013)^83^	https://github.com/alexdobin/STAR
String (v.12)	Global Biodata Coalition and ELIXIR	https://string-db.org/
EHM contraction analysis: Myrimager prototype software	N/A	N/A
Seahorse Wave Desktop Software	Agilent	https://www.agilent.com/en/product/cell-analysis/real-time-cell-metabolic-analysis/xf-software/seahorse-wave-desktop-software-740897

### Experimental model and study participant details

The work presented here fulfills Dutch criteria of the code of proper use of human tissue. Data obtained from explanted human hearts of which the collection in the biobank was approved by the scientific advisory board of the biobank of the University Medical Center Utrecht, the Netherlands (protocol no. 12-387, 14-513 and 15-252). Informed consent was obtained or in certain cases waived by the ethics committee when obtaining informed consent was not possible due to death of the patient. We obtained left and right ventricular tissue from the cardiac free wall of explanted human hearts for immunohistochemistry and immunoblot analyses. Briefly, samples were boiled for 20 minutes in EDTA (10 mM Tris, 1 mM EDTA and 0.05% Tween20 at pH 9.0) for DSP and BNIP3L, and in sodium citrate (10 mM sodium citrate at pH 6.0) for SOD2, followed by overnight incubation at 4°C with the primary antibody. The sections were then incubated with BrightVision Poly-AP anti-mouse/rabbit secondary antibody (Immunologic, Duiven, the Netherlands) and counterstained with hematoxylin. Chromogen Liquid Permanent Red (Agilent Dako, Santa Clara, CA) and the NanoZoomer (Hitachi, Hamamatsu, Japan) system were used for visualization. NDP.view 2 software was used for image processing. For immunoblotting, 25-50 μg of protein was used as input.

The clinical features of the patient bearing the *DSP* ^p.Lys569X/WT^ mutation are summarized on Table S1.

The *DSP*^p.Arg1113X/WT^ hiPSC line originated from the bone marrow CD34^+^ cells isolated from a healthy, Caucasian 31-year old individual (male).

### Method details

#### Spatial transcriptomics

The procedure followed has previously been described in Boogerd et al., and Junker et al., (Boogerd et al. 2022; Junker et al. 2014). Briefly, we obtained blocks (3 mm width and height) from the left ventricular wall of an explanted heart (*DSP* ^*p.Lys569X/WT*^) spanning the epi-to endocardium regions. This block was cryosectioned at 100 μm thickness. RNA was isolated from each section, followed by paired-end sequencing at 50 bp read length on an Illumina HiSeq 2500 system (Illumina, Hayward, CA). One hundred and fifty-nine sections passed quality control; tables with transcript counts were loaded into R (version 4.1.0). Subsequently, the percentage of mitochondrial reads was determined per slice (Figure S2A). Then, the mitochondrial reads and ERCC spike-in reads were excluded from further analysis, as were slices with a total read count of less than 3000 (Figures S2B and S2C). In addition, genes that did not show a read count higher than 5 in at least 3 slices were also excluded from further analysis. The transcript counts were normalized by dividing them by the total amount of reads of the corresponding slice, and multiplying them with the median of, where is the total amount of reads R in a slice s. Pair-wise correlations between transcriptomic profiles of slices were determined using the cor function from the R stats package based on the normalized expression and plotted in heatmaps (Figure S2D). In addition, Z-scores were determined for the gene expression values by subtraction of that gene’s mean expression and division by its standard deviation.

#### mRNA sequencing

For transcriptomic analysis, mRNA sequencing was carried out on one-month-old hiPSC-CMs. RNA libraries were constructed utilizing the TruSeq Stranded mRNA polyA kit (Illumina) following the instructions provided by the manufacturer. The NextSeq 2000 platform was used for sequencing, employing a paired-end sequencing approach with read lengths of 50 base pairs for each end. The sequencing run generated a total of 400 million reads, providing a substantial amount of data for downstream analysis. Quality control of the reads was conducted with FastQC, and alignment to the human genome (assembly GRCh37) was performed using STAR (version 2.7.8a). Differential gene expression was assessed with DESeq2 (version 1.2) employing pooled dispersion estimates. Further functional enrichment of differentially expressed genes was analyzed using the STRING database (version 11.5), with Homo sapiens as the background. The analysis focused on the enrichment of molecular function (GO-MF), biological process (GO-BP), cellular component (GO-CC), and KEGG pathways, adhering to the default settings provided by the database.

#### Genome targeting with CRISPR/Cas9

A detailed description regarding the introduction of a precise edit using CRISPR/Cas9 has previously been described in Kohela et al., (Kohela et al. 2021). Briefly, sgRNA were selected with the CCTop tool and cloned into the pSpCas9(BB)-2A-GFP vector (Addgene, Watertown, MA; Labuhn et al., 2018; Stemmer et al., 2015). Editing potential of each sgRNA was tested in HEK293T cells and quantified with a T7-endonuclease assay. The most suitable sgRNA was selected based on the level of editing and its proximity to the mutation-of-interest. Next, we targeted commercially available healthy hiPSCs as described before using the single-stranded oligodeoxynucleotide listed in Table S4. Successfully edited clones were identified by targeted amplification of the genomic region-of-interest using GoTaq® Green Master Mix (Promega, Madison, WI) and the primers listed in key resources table, followed by sequencing (Macrogen, Amsterdam, the Netherlands).

#### Off-targets

The three exonic or intronic off-target sites with the highest predicted off-target editing potential (CCTop tool) were PCR amplified using GoTaq® Green Master Mix and the primers listed in key resources table, followed by Sanger sequencing.

#### Karyo-sequencing

One-thousand hiPSCs were collected as a pellet and treated as follows: 10 μg of Proteinase K (NEB, Ipswich, MA) in 1x CutSmart Buffer (NEB, Ipswich, MA) was added to the cell pellet and incubated for 2 hours at 55°C followed by 10 minutes at 80°C. 10 μL of 10 U NLAIII (NEB, Ipswich, MA) in 1x CutSmart Buffer was added to the DNA and incubated for 2 hours at 37°C followed by 20 minutes at 80°C. Fragments were ligated to adapters by adding 20 μL of 800 U T4 DNA ligase (NEB, Ipswich, MA), 1 mM ATP (ThermoFisher Scientific, Waltham, MA) and 50 nM adapter in 1x T4 DNA ligase buffer. Mixture was incubated overnight at 16°C. Library preparation, sequencing and analysis was performed as described previously (Bolhaqueiro et al. 2019).

#### hiPSC cell culture conditions and directed differentiation

hiPSCs were cultured on Geltrex™ LDEV-Free, hESC-Qualified, Reduced Growth Factor Basement Membrane Matrix-coated wells (GIBCO, Grand Island, NY). Cells were refreshed with Essential 8™ Medium (GIBCO, Waltham, MA) on a daily basis. hiPSCs were passaged once they reached 80-100% confluency. Briefly, medium was aspirated and TrypLE Express Enzyme (GIBCO, Waltham, MA) was added for 5 minutes at 37°C. After incubation, 4 mL of Essential 8™ Medium, supplemented with 1 μM thiazovivin (Sigma-Aldrich, Saint Louis, MI), was added to the dissociated cells and transferred to a 15 mL Falcon tube. Cells were centrifuged for 3 minutes at 200 RCF. Subsequently, cells were seeded at a density of 15,000 cells/cm^2^ in Essential 8™ Medium, supplemented with 1 μM thiazovivin. Medium was refreshed the next day with plain Essential 8™. For directed differentiation towards cardiomyocytes, hiPSCs were grown until a 80-90% confluency was reached (Day 0). Medium was aspirated and cells were washed once with dPBS (GIBCO, Waltham, MA). Next, cells were fed with RPMI-1640-Medium-GlutaMAX™Supplement-HEPES (GIBCO, Waltham, MA) supplemented with 0.5 mg/mL human recombinant albumin (Sigma-Aldrich, Saint Louis, MI), 0.2 mg/mL L-Ascorbic Acid 2-Phosphate (Sigma-Aldrich, Saint Louis, MI), and 4 μM CHIR99021 (Sigma-Aldrich, Saint Louis, MI). After 48 hours (Day 2), medium was aspirated and cells were washed once with RPMI-1640-Medium-GlutaMAX™Supplement-HEPES, followed by addition of RPMI-1640-Medium-GlutaMAX™Supplement-HEPES supplemented with 0.5 mg/mL human recombinant albumin (Sigma-Aldrich, Saint Louis, MI), 0.2 mg/mL L-Ascorbic Acid 2-Phosphate, and 5 μM IWP2 (Sigma-Aldrich, Saint Louis, MI). On day 4 and day 6, cells were refreshed with RPMI-1640-Medium-GlutaMAX™Supplement-HEPES supplemented with 0.5 mg/mL human recombinant albumin and 0.2 mg/mL L-Ascorbic Acid 2-Phosphate. From day 8 onwards, cells were refresh every 3-4 days with RPMI-1640-Medium-GlutaMAX™-Supplement-HEPES supplemented with B-27™Supplement (50x)-serum free (GIBCO, Waltham, MA). hiPSC-derived cardiomyocytes were subsequently dissociated with TrypLE™ Select Enzyme (10x) without phenol red (GIBCO, Waltham, MA) for a maximum of 45 minutes at 37°C. Cardiomyocytes were seeded at a density of 100,000 cells/cm^2^ in Geltrex-coated wells for downstream molecular applications. All experiments were performed on 1-month old hiPSC-CMs.

#### Generation of the *DSP*^p.Arg1113X/WT^ line

We used CRISPR/Cas9 in combination with two single-stranded DNA templates to introduce a nonsense mutation in DSP in a control hiPSC line in a heterozygous manner (DSP c.3337 C>T; DSP p.Arg1113X; Figure S5A; Table S3). In addition to the desired edit, an additional synonymous mutation (blocking mutation) was incorporated into the genome to prevent Cas9 from recutting (Figure S5B). Guides were selected based on results from a T7 endonuclease I (T7E1) assay, allowing for the identification of sequences with effective cleavage activity and efficient target recognition (Figure S5C). The *DSP* ^p.Arg1113X/WT^ hiPSC line showed uniform expression of the pluripotency markers nanog homeobox (NANOG), POU class 5 homeobox 1 (OCT3/4) and SRY-Box transcription factor 2 (SOX2; Figure S5D). Moreover, karyo-sequencing revealed no chromosomal aberrations and no editing events were detected in the top three predicted off-target sites; peptidylprolyl Isomerase E (*PPIE*), coiled-coil domain containing 85A (*CCDC85A*) and contactin 5 (*CNTN5*; Figures S5E and S5F).

#### Immunocytochemistry

hiPSC-derived cardiomyocytes were seeded on Geltrex-coated glass coverslips with a diameter of 12 mm. Seven days later, cells were washed once with dPBS and then fixed with 2% paraformaldehyde for 30 minutes at room temperature. Cardiomyocytes were washed 3x with dPBS and permeabilized with 0.1% Triton X-100 in dPBS for 8 minutes at room temperature. Permeabilized cells were blocked with 4% goat serum (ThermoFisher Scientific, Waltham, MA) in dPBS for 1 hour at room temperature. Cells were then incubated with primary antibody diluted in 4% goat serum solution and incubated overnight at 4°C. Next, cells were washed 3x times with dPBS and incubated with the corresponding secondary antibody dissolved in 4% goat serum solution for 1 hour at room temperature. Nuclei were stained with 3 μM DAPI (ThermoFisher Scientific, Waltham, MA) for 5 minutes at room temperature. Cells were mounted with Mowiol (24% (w/v) Glycerol (Baker, Phillipsburg, NJ), 9.6% (w/v) Mowiol 4-88 (Calbiochem, San Diego, CA), 0.1 M Tris-HCl pH 8.5) and imaged using a Leica TCS SPE Confocal Microscope (Leica, Wetzlar, Germany). Antibodies and the dilutions used can be found in key resources table.

#### Immunohistochemistry

Patient ACM samples were fixed in 4% paraformaldehyde prior to paraffin embedding. Masson’s trichrome staining was performed for identification of fibrofatty replacement areas. For immunohistochemistry, antigen retrieval was achieved using the appropriate antigen retrieval method EDTA (10 mM Tris, 1 mM EDTA and 0.05% TWEEN20 at pH9) or sodium citrate (10 mM sodium citrate at pH6, or pepsin digestion (107185, Sigma Aldrich). The slides were subjected to the selected primary antibody prior to incubation with the appropriate secondary antibody, BrightVision Poly-AP anti-mouse or -rabbit antibody conjugate (Immunologic). Visualization of the signal was achieved using Chromogen Liquid Permanent Red (Dako) and slides were counterstained with hematoxylin. Microscopic slides were digitalized using a NanoZoomer (Hitachi) scanner and processed with NDP view software.

#### Immunoblotting

hiPSC-derived cardiomyocytes were dissociated with TrypLE™ Select Enzyme (10x) and collected in a 1.5 mL Eppendorf tube. Cells were centrifuged for 5 minutes at 300 RCF. Supernatant was removed and cells were resuspended in 1 mL dPBS followed by centrifugation at 300 RCF for 5 minutes. Cells were lysed in RIPA buffer (50 mM Tris-pH7.5, 150 mM NaCl, 0.1% SDS, 0.5% sodium deoxycholate, 1% Triton X-100) supplemented with 1 tablet of cOmplete™ EDTA-free Protease Inhibitor Cocktail (Roche, Basel, Switzerland) and 1 tablet of PhosSTOP™ (Roche, Basel, Switzerland) per 10 mL of RIPA buffer. Immunoblotting was performed using 10-15 μg of protein extract. For detection, the corresponding secondary antibody coupled to horseradish peroxidase was used in combination with the Clarity™ Western ECL Substrate kit (Bio Rad, Hercules, CA). Immunoblots were imaged with an Amersham Imager 680RGB device (GE Healthcare, Chicago, IL) and quantified with ImageJ. Antibodies and the dilutions used can be found in key resources table. Control group was set at one.

#### Quantitative PCR

Total RNA was isolated from hiPSC-derived cardiomyocytes using the RNeasy Mini Kit (Qiagen, Hilden, Germany) following the protocol supplied by the manufacturer. Total RNA was reverse transcribed using the iScript™ cDNA Synthesis Kit (Bio Rad, Hercules, CA). Quantitative PCR (qPCR) reactions were performed on a Bio Rad CFX96 Real-Time PCR Detection System using the iQ SYBR Green Supermix kit (Bio Rad, Hercules, CA). Primers used for amplification are listed in key resources table. The ΔΔCt-method was used to analyze the data. Control group was set at one.

#### Seahorse assay

hiPSC-derived cardiomyocytes were plated at 60,000 cells per well in a 24-well cell culture microplate (Agilent, Santa Clara, CA). One week after plating, the cells were incubated with Seahorse XF base medium (Agilent, Santa Clara, CA) supplemented with 10 mM glucose, 1 mM pyruvate, 2 mM glutamine for 30 minutes before running the XF Cell Mito Stress Test (Agilent, Santa Clara, CA; Oligomycin 1 μM, FCCP 4 μM, Rotenone and Antimycin A 2.5 μM) on a XFe24 Analyzer (Agilent, Santa Clara, CA). For normalization, the CyQUANT Cell Proliferation Assay (ThermoFisher Scientific, Waltham, MA) was used according to the manufacture’s protocol and the data was analyzed using the Seahorse Wave Desktop Software (Agilent, Santa Clara, CA).

#### Reactive oxygen species (ROS) quantification

hiPSC-CMs were cultured under standard conditions and subsequently incubated with 10 μM 2',7'-dichlorodihydrofluorescein diacetate (H2DCFDA; Invitrogen, D399) for 30 minutes at 37°C in the dark. N-acetylcysteine (NAC; Sigma-Aldrich, 1124220025) was used as a negative control to validate the specificity of ROS detection. Cells were pretreated with 5 mM NAC for 1 hour prior to H2DCFDA addition to mitigate ROS levels. After incubation, cells were washed three times with dPBS (GIBCO, Waltham, MA) to remove excess dye, and fluorescence microscopy was performed using appropriate filters for DCF detection (excitation/emission: 495/529 nm).

Quantification of ROS was performed using ImageJ software. Fluorescent images were converted to grayscale, and background fluorescence was subtracted. Regions of interest (ROIs) encompassing individual cells were defined, and the mean fluorescence intensity (MFI) for each ROI was measured. Data were normalized to control samples without NAC treatment to quantify relative ROS levels.

#### Knockdown experiments

Three-week-old iPSC-CMs were dissociated using TrypLE™ Select Enzyme (10x), no phenol red and seeded at a cell density of 100,000 cells/cm^2^ in Geltrex-coated wells with RPMI-1640-Medium-GlutaMAX™Supplement-HEPES (GIBCO, Waltham, MA) supplemented with B-27™ Supplement (50x)-serum free (GIBCO, Waltham, MA) and 2 μM thiazovivin (Sigma-Aldrich, Saint Louis, MI). After 24 hours, medium was refreshed with RPMI-1640-Medium-GlutaMAX™Supplement-HEPES supplemented with B-27™ Supplement (50x)-serum free. Seven days after reseeding, cells were transfected with either 10 nM scramble or siRNA oligo duplexes targeting *DSP* or *EPAS1* (OriGene, Rockville, MD) utilizing Lipofectamine™ RNAiMAX (ThermoFisher Scientific, Waltham, MA) according to the manufacturers’ instructions. After 48 hours, RNA was isolated (as described above) and protein samples were collected after 72 hours (as described above).

#### TUNEL staining

The differentiated cardiomyocytes were seeded on Geltrex-coated glass coverslips with a diameter of 12 mm. After 7 days, the cells were washed twice with dPBS and then fixed with 2% paraformaldehyde for 30 minutes at room temperature. The cardiomyocytes were washed three times with dPBS and permeabilized with 0.1% Triton X-100 in dPBS for 8 minutes at room temperature. Subsequently, the coverslips were incubated on ice in pre-chilled permeabilization solution (0.1% Triton X-100 and 0.1% sodium citrate in distilled water) for 2 minutes. The coverslips were then rinsed twice in dPBS for 5 minutes each. Next, the coverslips were incubated with TUNEL reaction mixture for 1 hour at 37°C in the dark, using a setup involving a tip box filled with warm water, parafilm on tip holders, and covered with aluminum foil to maintain the dark environment in the water bath. Following the TUNEL reaction, the coverslips were blocked in 4% goat serum for 1 hour.

#### EHM generation

EHM was generated according to the protocol published by Tiburcy et al., 2017.^48^ In brief, patient-derived iPS-cell-derived CMs (purity >90%) were mixed together with HFFs (HFF-1, ATCC, SCRC-1041) at a ratio of 70:30. The cell mixture was resuspended in an appropriate volume of Collagen type I (Collagen Solutions, FS22024) diluted into RPMI 2× (Thermo Fisher Scientific, 51800-035). A total of 185 μl of the cell–collagen mixture was cast in each well of a 48 EHM multi-well plate (myrPlate-TM5; myriamed GmbH). The cast mixture was incubated for approximately 45 min at 37°C and subsequently EHM medium freshly supplemented with TGFβ1 (Peprotech, AF-100-21C) was added. During the initial 3 days following the casting process, tissue medium was refreshed daily with EHM medium supplemented with TGFβ1. Subsequently, the tissue medium was replaced daily with EHM medium for the entirety of the experimental duration.

#### Contraction analyses

Contraction measurements were performed using video-optic recordings of EHM mediated pole bending in a myrPlate-TM5 culture format at 37°C (ref.^84^). Data were recorded from spontaneously contraction EHM for at least 2 min at 50 fps at the indicated time points in a myrImager prototype (myriamed GmbH). Percent pole bending is reported as a surrogate for force of contraction (*F*); contraction and relaxation times are recorded from 20% to 80% peak contraction and 20% to 80% relaxation; contraction and relaxation velocities are reported as maximal and mininmal d*F*/d*t*.

### Quantification and statistical analysis

The number of independent cardiomyocyte batches and technical replicates within each differentiation are indicated in the figure legends. All data presented as mean. Statistics have been performed with PRISM (GraphPad Software Inc. version 9). Unless otherwise noted, datasets were first checked for outliers using the ROUT method (Q = 5%) and were removed if present. Secondly, data were tested for normality using the Kolmogorov-Smirnov test with Dallal-Wilkinson-Lillie for *p*-value method (alpha = 0.05). Significance between two groups was assessed by a two-tailed unpaired Student’s t-test or two-tailed Mann-Whitney test when data were not normally distributed. Significance and the degree of correlation between the expression level of two proteins has been assessed by non-parametric Spearman’s correlation (two-tailed; alpha = 0.05). Data was considered significantly different when *p*<0.05. Asterisks in the figures indicate the degree of significance (∗ = *p* < 0.05, ∗∗ = *p* < 0.01, ∗∗∗ = *p* < 0.001, ∗∗∗∗ = *p* < 0.0001).

## Supplemental information

Document S1. Figures S1–S6 and Tables S1 and S3–S7

Table S2. Excel file containing all differentially expressed genes within cluster 4, related to Figure 1

## References

[1] Boogerd C.J., Lacraz G.P.A., Vértesy Á., van Kampen S.J., Perini I., de Ruiter H., Versteeg D., Brodehl A., van der Kraak P., Giacca M. (2023). Spatial transcriptomics unveils ZBTB11 as a regulator of cardiomyocyte degeneration in arrhythmogenic cardiomyopathy. Cardiovasc. Res..

[2] Hoorntje E.T., Te Rijdt W.P., James C.A., Pilichou K., Basso C., Judge D.P., Bezzina C.R., van Tintelen J.P. (2017). Arrhythmogenic cardiomyopathy: pathology, genetics, and concepts in pathogenesis. Cardiovasc. Res..

[3] James C.A., Bhonsale A., Tichnell C., Murray B., Russell S.D., Tandri H., Tedford R.J., Judge D.P., Calkins H. (2013). Exercise increases age-related penetrance and arrhythmic risk in arrhythmogenic right ventricular dysplasia/cardiomyopathy-associated desmosomal mutation carriers. J. Am. Coll. Cardiol..

[4] Tanawuttiwat T., Sager S.J., Hare J.M., Myerburg R.J. (2013). Myocarditis and ARVC/D: variants or mimics?. Heart Rhythm.

[5] Delmar M., McKenna W.J. (2010). The cardiac desmosome and arrhythmogenic cardiomyopathies: from gene to disease. Circ. Res..

[6] Vermij S.H., Abriel H., van Veen T.A.B. (2017). Refining the molecular organization of the cardiac intercalated disc. Cardiovasc. Res..

[7] Austin K.M., Trembley M.A., Chandler S.F., Sanders S.P., Saffitz J.E., Abrams D.J., Pu W.T. (2019). Molecular mechanisms of arrhythmogenic cardiomyopathy. Nat. Rev. Cardiol..

[8] Jansen J.A., Noorman M., Musa H., Stein M., de Jong S., van der Nagel R., Hund T.J., Mohler P.J., Vos M.A., van Veen T.A. (2012). Reduced heterogeneous expression of Cx43 results in decreased Nav1.5 expression and reduced sodium current that accounts for arrhythmia vulnerability in conditional Cx43 knockout mice. Heart Rhythm.

[9] Veeraraghavan R., Gourdie R.G. (2016). STORM-based Quantitative Assessment of Sodium Channel Localization Relative to Junctional Proteins Within the Cardiac Intercalated Disk. Microsc. Microanal..

[10] Kim J.C., Pérez-Hernández M., Alvarado F.J., Maurya S.R., Montnach J., Yin Y., Zhang M., Lin X., Vasquez C., Heguy A. (2019). Disruption of Ca(2+)(i) Homeostasis and Connexin 43 Hemichannel Function in the Right Ventricle Precedes Overt Arrhythmogenic Cardiomyopathy in Plakophilin-2-Deficient Mice. Circulation.

[11] Sato P.Y., Musa H., Coombs W., Guerrero-Serna G., Patiño G.A., Taffet S.M., Isom L.L., Delmar M. (2009). Loss of plakophilin-2 expression leads to decreased sodium current and slower conduction velocity in cultured cardiac myocytes. Circ. Res..

[12] Cerrone M., Noorman M., Lin X., Chkourko H., Liang F.X., van der Nagel R., Hund T., Birchmeier W., Mohler P., van Veen T.A. (2012). Sodium current deficit and arrhythmogenesis in a murine model of plakophilin-2 haploinsufficiency. Cardiovasc. Res..

[13] Oxford E.M., Musa H., Maass K., Coombs W., Taffet S.M., Delmar M. (2007). Connexin43 remodeling caused by inhibition of plakophilin-2 expression in cardiac cells. Circ. Res..

[14] Kam C.Y., Dubash A.D., Magistrati E., Polo S., Satchell K.J.F., Sheikh F., Lampe P.D., Green K.J. (2018). Desmoplakin maintains gap junctions by inhibiting Ras/MAPK and lysosomal degradation of connexin-43. J. Cell Biol..

[15] van Kampen S.J., Han S.J., van Ham W.B., Kyriakopoulou E., Stouthart E.W., Goversen B., Monshouwer-Kloots J., Perini I., de Ruiter H., van der Kraak P. (2023). PITX2 induction leads to impaired cardiomyocyte function in arrhythmogenic cardiomyopathy. Stem Cell Rep..

[16] Asatryan B., Asimaki A., Landstrom A.P., Khanji M.Y., Odening K.E., Cooper L.T., Marchlinski F.E., Gelzer A.R., Semsarian C., Reichlin T. (2021). Inflammation and Immune Response in Arrhythmogenic Cardiomyopathy: State-of-the-Art Review. Circulation.

[17] Pérez-Hernández M., Marrón-Liñares G.M., Schlamp F., Heguy A., van Opbergen C.J.M., Mezzano V., Zhang M., Liang F.X., Cerrone M., Delmar M. (2020). Transcriptomic Coupling of PKP2 With Inflammatory and Immune Pathways Endogenous to Adult Cardiac Myocytes. Front. Physiol..

[18] Garcia-Gras E., Lombardi R., Giocondo M.J., Willerson J.T., Schneider M.D., Khoury D.S., Marian A.J. (2006). Suppression of canonical Wnt/beta-catenin signaling by nuclear plakoglobin recapitulates phenotype of arrhythmogenic right ventricular cardiomyopathy. J. Clin. Invest..

[19] Smith E.D., Lakdawala N.K., Papoutsidakis N., Aubert G., Mazzanti A., McCanta A.C., Agarwal P.P., Arscott P., Dellefave-Castillo L.M., Vorovich E.E. (2020). Desmoplakin Cardiomyopathy, a Fibrotic and Inflammatory Form of Cardiomyopathy Distinct From Typical Dilated or Arrhythmogenic Right Ventricular Cardiomyopathy. Circulation.

[20] Yang Z., Bowles N.E., Scherer S.E., Taylor M.D., Kearney D.L., Ge S., Nadvoretskiy V.V., DeFreitas G., Carabello B., Brandon L.I. (2006). Desmosomal dysfunction due to mutations in desmoplakin causes arrhythmogenic right ventricular dysplasia/cardiomyopathy. Circ. Res..

[21] Martherus R., Jain R., Takagi K., Mendsaikhan U., Turdi S., Osinska H., James J.F., Kramer K., Purevjav E., Towbin J.A. (2016). Accelerated cardiac remodeling in desmoplakin transgenic mice in response to endurance exercise is associated with perturbed Wnt/β-catenin signaling. Am. J. Physiol. Heart Circ. Physiol..

[22] Gomes J., Finlay M., Ahmed A.K., Ciaccio E.J., Asimaki A., Saffitz J.E., Quarta G., Nobles M., Syrris P., Chaubey S. (2012). Electrophysiological abnormalities precede overt structural changes in arrhythmogenic right ventricular cardiomyopathy due to mutations in desmoplakin-A combined murine and human study. Eur. Heart J..

[23] Lyon R.C., Mezzano V., Wright A.T., Pfeiffer E., Chuang J., Banares K., Castaneda A., Ouyang K., Cui L., Contu R. (2014). Connexin defects underlie arrhythmogenic right ventricular cardiomyopathy in a novel mouse model. Hum. Mol. Genet..

[24] Gusev K., Khudiakov A., Zaytseva A., Perepelina K., Makeenok S., Kaznacheyeva E., Kostareva A. (2020). Impact of the DSP-H1684R Genetic Variant on Ion Channels Activity in iPSC-Derived Cardiomyocytes. Cell. Physiol. Biochem..

[25] Pérez-Hernández M., van Opbergen C.J.M., Bagwan N., Vissing C.R., Marrón-Liñares G.M., Zhang M., Torres Vega E., Sorrentino A., Drici L., Sulek K. (2022). Loss of Nuclear Envelope Integrity and Increased Oxidant Production Cause DNA Damage in Adult Hearts Deficient in PKP2: A Molecular Substrate of ARVC. Circulation.

[26] van Opbergen C.J.M., den Braven L., Delmar M., van Veen T.A.B. (2019). Mitochondrial Dysfunction as Substrate for Arrhythmogenic Cardiomyopathy: A Search for New Disease Mechanisms. Front. Physiol..

[27] Volani C., Medici A., Philippe R., Lavdas A., Della Corte I., Lang M., Frommelt L.S., Blumer M., Sommariva E., Pompilio G. (2024). Mitochondrial players as novel therapeutic options in arrhythmogenic cardiomyopathy. Cardiovasc. Res..

[28] Lippi M., Maione A.S., Chiesa M., Perrucci G.L., Iengo L., Sattin T., Cencioni C., Savoia M., Zeiher A.M., Tundo F. (2023). Omics Analyses of Stromal Cells from ACM Patients Reveal Alterations in Chromatin Organization and Mitochondrial Homeostasis. Int. J. Mol. Sci..

[29] Tsutsui H., Kinugawa S., Matsushima S. (2011). Oxidative stress and heart failure. Am. J. Physiol. Heart Circ. Physiol..

[30] Sovari A.A. (2016). Cellular and Molecular Mechanisms of Arrhythmia by Oxidative Stress. Cardiol. Res. Pract..

[31] Kruse F., Junker J.P., van Oudenaarden A., Bakkers J. (2016). Tomo-seq: A method to obtain genome-wide expression data with spatial resolution. Methods Cell Biol..

[32] Kishimoto I., Rossi K., Garbers D.L. (2001). A genetic model provides evidence that the receptor for atrial natriuretic peptide (guanylyl cyclase-A) inhibits cardiac ventricular myocyte hypertrophy. Proc. Natl. Acad. Sci. USA.

[33] Nagata Y., Yamagishi M., Konno T., Nakanishi C., Asano Y., Ito S., Nakajima Y., Seguchi O., Fujino N., Kawashiri M.A. (2017). Heart Failure Phenotypes Induced by Knockdown of DAPIT in Zebrafish: A New Insight into Mechanism of Dilated Cardiomyopathy. Sci. Rep..

[34] Wang X., Su H., Ranek M.J. (2008). Protein quality control and degradation in cardiomyocytes. J. Mol. Cell. Cardiol..

[35] Fernandez-Caggiano M., Kamynina A., Francois A.A., Prysyazhna O., Eykyn T.R., Krasemann S., Crespo-Leiro M.G., Vieites M.G., Bianchi K., Morales V. (2020). Mitochondrial pyruvate carrier abundance mediates pathological cardiac hypertrophy. Nat. Metab..

[36] Wu A., Zhong C., Song X., Yuan W., Tang M., Shu T., Huang H., Yang P., Liu Q. (2024). The activation of LBH-CRYAB signaling promotes cardiac protection against I/R injury by inhibiting apoptosis and ferroptosis. iScience.

[37] Ghosh R., Pattison J.S. (2018). Macroautophagy and Chaperone-Mediated Autophagy in Heart Failure: The Known and the Unknown. Oxid. Med. Cell. Longev..

[38] Voors A.A., Kremer D., Geven C., Ter Maaten J.M., Struck J., Bergmann A., Pickkers P., Metra M., Mebazaa A., Düngen H.D., Butler J. (2019). Adrenomedullin in heart failure: pathophysiology and therapeutic application. Eur. J. Heart Fail..

[39] Oktay Y., Dioum E., Matsuzaki S., Ding K., Yan L.J., Haller R.G., Szweda L.I., Garcia J.A. (2007). Hypoxia-inducible factor 2alpha regulates expression of the mitochondrial aconitase chaperone protein frataxin. J. Biol. Chem..

[40] Li Y., Zheng W., Lu Y., Zheng Y., Pan L., Wu X., Yuan Y., Shen Z., Ma S., Zhang X. (2021). BNIP3L/NIX-mediated mitophagy: molecular mechanisms and implications for human disease. Cell Death Dis..

[41] Heinz S., Benner C., Spann N., Bertolino E., Lin Y.C., Laslo P., Cheng J.X., Murre C., Singh H., Glass C.K. (2010). Simple combinations of lineage-determining transcription factors prime cis-regulatory elements required for macrophage and B cell identities. Mol. Cell.

[42] Semenza G.L. (2012). Hypoxia-inducible factors in physiology and medicine. Cell.

[43] Basso C., Pilichou K., Bauce B., Corrado D., Thiene G. (2018). Diagnostic Criteria, Genetics, and Molecular Basis of Arrhythmogenic Cardiomyopathy. Heart Fail. Clin..

[44] Haas J., Frese K.S., Peil B., Kloos W., Keller A., Nietsch R., Feng Z., Müller S., Kayvanpour E., Vogel B. (2015). Atlas of the clinical genetics of human dilated cardiomyopathy. Eur. Heart J..

[45] Elliott P., O'Mahony C., Syrris P., Evans A., Rivera Sorensen C., Sheppard M.N., Carr-White G., Pantazis A., McKenna W.J. (2010). Prevalence of desmosomal protein gene mutations in patients with dilated cardiomyopathy. Circ. Cardiovasc. Genet..

[46] Muñoz-Sánchez J., Chánez-Cárdenas M.E. (2019). The use of cobalt chloride as a chemical hypoxia model. J. Appl. Toxicol..

[47] Chen J. (2016). The Cell-Cycle Arrest and Apoptotic Functions of p53 in Tumor Initiation and Progression. Cold Spring Harb. Perspect. Med..

[48] Tiburcy M., Hudson J.E., Balfanz P., Schlick S., Meyer T., Chang Liao M.L., Levent E., Raad F., Zeidler S., Wingender E. (2017). Defined Engineered Human Myocardium With Advanced Maturation for Applications in Heart Failure Modeling and Repair. Circulation.

[49] Corrado D., Wichter T., Link M.S., Hauer R.N.W., Marchlinski F.E., Anastasakis A., Bauce B., Basso C., Brunckhorst C., Tsatsopoulou A. (2015). Treatment of Arrhythmogenic Right Ventricular Cardiomyopathy/Dysplasia. Circulation.

[50] Lee P., Chandel N.S., Simon M.C. (2020). Cellular adaptation to hypoxia through hypoxia inducible factors and beyond. Nat. Rev. Mol. Cell Biol..

[51] Nauta T.D., van den Broek M., Gibbs S., van der Pouw-Kraan T.C.T.M., Oudejans C.B., van Hinsbergh V.W.M., Koolwijk P. (2017). Identification of HIF-2α-regulated genes that play a role in human microvascular endothelial sprouting during prolonged hypoxia *in vitro*. Angiogenesis.

[52] Downes N.L., Laham-Karam N., Kaikkonen M.U., Ylä-Herttuala S. (2018). Differential but Complementary HIF1α and HIF2α Transcriptional Regulation. Mol. Ther..

[53] Diebold I., Petry A., Hess J., Görlach A. (2010). The NADPH oxidase subunit NOX4 is a new target gene of the hypoxia-inducible factor-1. Mol. Biol. Cell.

[54] Schweers R.L., Zhang J., Randall M.S., Loyd M.R., Li W., Dorsey F.C., Kundu M., Opferman J.T., Cleveland J.L., Miller J.L., Ney P.A. (2007). NIX is required for programmed mitochondrial clearance during reticulocyte maturation. Proc. Natl. Acad. Sci. USA.

[55] Sandoval H., Thiagarajan P., Dasgupta S.K., Schumacher A., Prchal J.T., Chen M., Wang J. (2008). Essential role for Nix in autophagic maturation of erythroid cells. Nature.

[56] Moyzis A., Gustafsson Å.B. (2019). Multiple recycling routes: Canonical vs. non-canonical mitophagy in the heart. Biochim. Biophys. Acta, Mol. Basis Dis..

[57] Vande Velde C., Cizeau J., Dubik D., Alimonti J., Brown T., Israels S., Hakem R., Greenberg A.H. (2000). BNIP3 and genetic control of necrosis-like cell death through the mitochondrial permeability transition pore. Mol. Cell Biol..

[58] Diwan A., Matkovich S.J., Yuan Q., Zhao W., Yatani A., Brown J.H., Molkentin J.D., Kranias E.G., Dorn G.W. (2009). Endoplasmic reticulum-mitochondria crosstalk in NIX-mediated murine cell death. J. Clin. Invest..

[59] Hamacher-Brady A., Choe S.C., Krijnse-Locker J., Brady N.R. (2014). Intramitochondrial recruitment of endolysosomes mediates Smac degradation and constitutes a novel intrinsic apoptosis antagonizing function of XIAP E3 ligase. Cell Death Differ..

[60] Yussman M.G., Toyokawa T., Odley A., Lynch R.A., Wu G., Colbert M.C., Aronow B.J., Lorenz J.N., Dorn G.W. (2002). Mitochondrial death protein Nix is induced in cardiac hypertrophy and triggers apoptotic cardiomyopathy. Nat. Med..

[61] Gálvez A.S., Brunskill E.W., Marreez Y., Benner B.J., Regula K.M., Kirschenbaum L.A., Dorn G.W. (2006). Distinct pathways regulate proapoptotic Nix and BNip3 in cardiac stress. J. Biol. Chem..

[62] Mallat Z., Tedgui A., Fontaliran F., Frank R., Durigon M., Fontaine G. (1996). Evidence of apoptosis in arrhythmogenic right ventricular dysplasia. N. Engl. J. Med..

[63] Pilichou K., Remme C.A., Basso C., Campian M.E., Rizzo S., Barnett P., Scicluna B.P., Bauce B., van den Hoff M.J.B., de Bakker J.M.T. (2009). Myocyte necrosis underlies progressive myocardial dystrophy in mouse dsg2-related arrhythmogenic right ventricular cardiomyopathy. J. Exp. Med..

[64] Flynn J.M., Melov S. (2013). SOD2 in mitochondrial dysfunction and neurodegeneration. Free Radic. Biol. Med..

[65] Li Y., Huang T.T., Carlson E.J., Melov S., Ursell P.C., Olson J.L., Noble L.J., Yoshimura M.P., Berger C., Chan P.H. (1995). Dilated cardiomyopathy and neonatal lethality in mutant mice lacking manganese superoxide dismutase. Nat. Genet..

[66] Kang P.T., Chen C.L., Ohanyan V., Luther D.J., Meszaros J.G., Chilian W.M., Chen Y.R. (2015). Overexpressing superoxide dismutase 2 induces a supernormal cardiac function by enhancing redox-dependent mitochondrial function and metabolic dilation. J. Mol. Cell. Cardiol..

[67] Shen X., Zheng S., Metreveli N.S., Epstein P.N. (2006). Protection of cardiac mitochondria by overexpression of MnSOD reduces diabetic cardiomyopathy. Diabetes.

[68] Semenza G.L. (2023). Hypoxia-inducible factors: roles in cardiovascular disease progression, prevention, and treatment. Cardiovasc. Res..

[69] Zhou S., Sun W., Zhang Z., Zheng Y. (2014). The role of Nrf2-mediated pathway in cardiac remodeling and heart failure. Oxid. Med. Cell. Longev..

[70] Chun Y., Kim J. (2021). AMPK-mTOR Signaling and Cellular Adaptations in Hypoxia. Int. J. Mol. Sci..

[71] Edmunds L.R., Sharma L., Wang H., Kang A., d'Souza S., Lu J., McLaughlin M., Dolezal J.M., Gao X., Weintraub S.T. (2015). c-Myc and AMPK Control Cellular Energy Levels by Cooperatively Regulating Mitochondrial Structure and Function. PLoS One.

[72] De Nicolo B., Cataldi-Stagetti E., Diquigiovanni C., Bonora E. (2023). Calcium and Reactive Oxygen Species Signaling Interplays in Cardiac Physiology and Pathologies. Antioxidants.

[73] Hölscher M., Silter M., Krull S., von Ahlen M., Hesse A., Schwartz P., Wielockx B., Breier G., Katschinski D.M., Zieseniss A. (2011). Cardiomyocyte-specific prolyl-4-hydroxylase domain 2 knock out protects from acute myocardial ischemic injury. J. Biol. Chem..

[74] Pradeep S.R., Lim S.T., Thirunavukkarasu M., Joshi M., Cernuda B., Palesty J.A., Maulik N. (2022). Protective Effect of Cardiomyocyte-Specific Prolyl-4-Hydroxylase 2 Inhibition on Ischemic Injury in a Mouse MI Model. J. Am. Coll. Surg..

[75] Ali S.R., Nguyen N.U.N., Menendez-Montes I., Hsu C.C., Elhelaly W., Lam N.T., Li S., Elnwasany A., Nakada Y., Thet S. (2024). Hypoxia-induced stabilization of HIF2A promotes cardiomyocyte proliferation by attenuating DNA damage. J. Cardiovasc. Aging.

[76] Pitsch M., Kant S., Mytzka C., Leube R.E., Krusche C.A. (2021). Autophagy and Endoplasmic Reticulum Stress during Onset and Progression of Arrhythmogenic Cardiomyopathy. Cells.

[77] Loiben A., Friedman C.E., Chien W.M., Stempien-Otero A., Lin S., Yang K.C. (2023). Generation of human iPSC line from an arrhythmogenic cardiomyopathy patient with a DSP protein-truncating variant. Stem Cell Res..

[78] Olcum M., Rouhi L., Fan S., Gonzales M.M., Jeong H.H., Zhao Z., Gurha P., Marian A.J. (2023). PANoptosis is a prominent feature of desmoplakin cardiomyopathy. J. Cardiovasc. Aging.

[79] Moslehi J., Minamishima Y.A., Shi J., Neuberg D., Charytan D.M., Padera R.F., Signoretti S., Liao R., Kaelin W.G. (2010). Loss of hypoxia-inducible factor prolyl hydroxylase activity in cardiomyocytes phenocopies ischemic cardiomyopathy. Circulation.

[80] May D., Gilon D., Djonov V., Itin A., Lazarus A., Gordon O., Rosenberger C., Keshet E. (2008). Transgenic system for conditional induction and rescue of chronic myocardial hibernation provides insights into genomic programs of hibernation. Proc. Natl. Acad. Sci. USA.

[81] Szyller J., Jagielski D., Bil-Lula I. (2022). Antioxidants in Arrhythmia Treatment-Still a Controversy? A Review of Selected Clinical and Laboratory Research. Antioxidants.

[82] Love M.I., Huber W., Anders S. (2014). Moderated estimation of fold change and dispersion for RNA-seq data with DESeq2. Genome Biol..

[83] Dobin A., Davis C.A., Schlesinger F., Drenkow J., Zaleski C., Jha S., Batut P., Chaisson M., Gingeras T.R. (2013). STAR: ultrafast universal RNA-seq aligner. Bioinformatics.

[84] Tiburcy M., Meyer T., Liaw N.Y., Zimmermann W.H. (2020). Generation of Engineered Human Myocardium in a Multi-well Format. STAR Protoc..

