# Supplementary material for: EPAS1 induction drives myocardial degeneration in desmoplakin-cardiomyopathy
Source: iScience. 2025 Jan 25;28(3):111895. doi: 10.1016/j.isci.2025.111895 (PMC11872638; doi:10.1016/j.isci.2025.111895)
Supplement: Document S1. Figures S1–S6 and Tables S1 and S3–S7 [file mmc1.pdf]

iScience, Volume *28*

Supplemental information

EPAS1 induction drives myocardial degeneration in desmoplakin-cardiomyopathy

Eirini Kyriakopoulou, Sebastiaan J. van Kampen, Martijn Wehrens, Su Ji Han, Hesther de Ruiter, Jantine Monshouwer-Kloots, Emma Marshall, Andreas Brodehl, Petra van der Kraak, Anneline S.J.M. te Riele, Egidius E.H.L. van Aarnhem, Linda W. van Laake, Hoyee Tsui, Cornelis J. Boogerd, and Eva van Rooij

Supplemental Files

Supplementary Figure 1

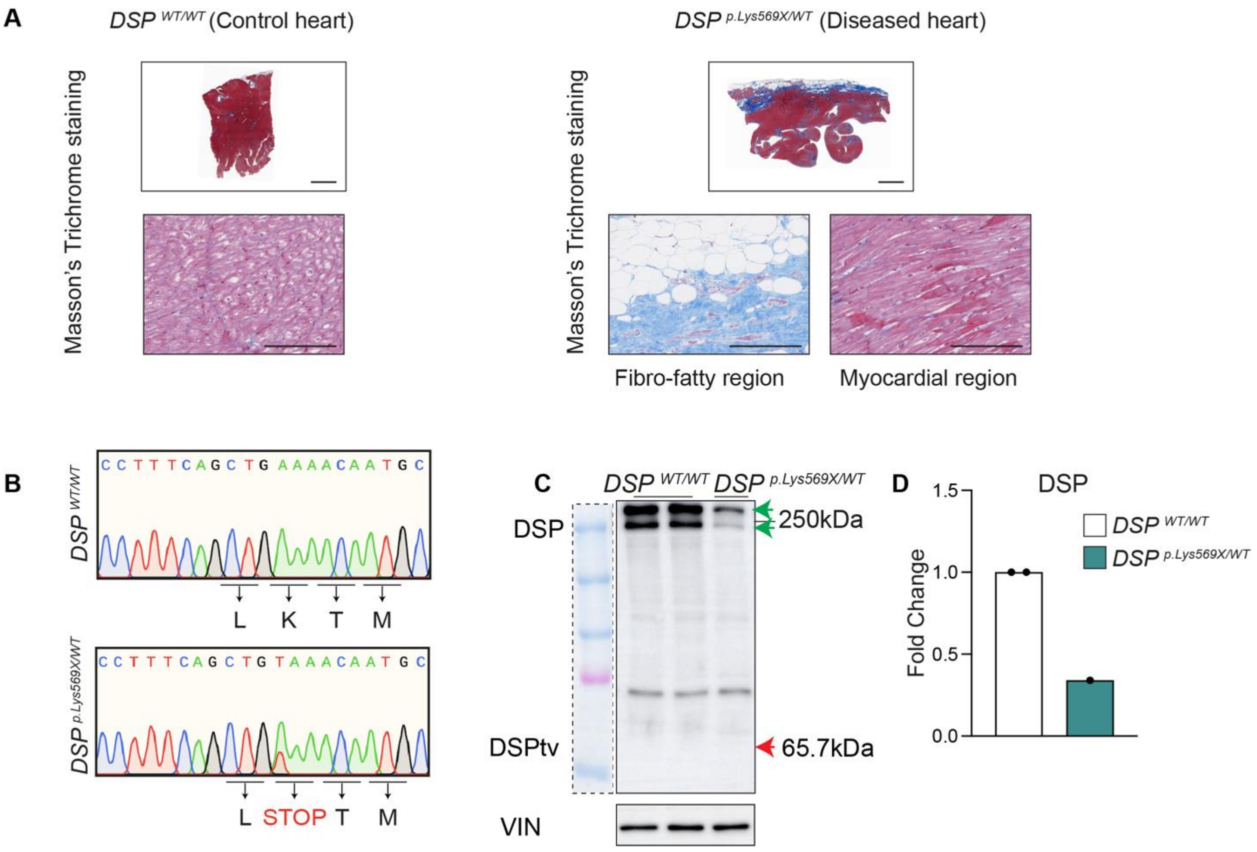

Figure S1. The pathogenic *DSP* p.Lys569X variant causes pathological cardiac remodeling.

(**A**) Masson's trichrome staining on transverse LV sections of a non-failing control heart (*DSP* $^{WT/WT}$) and the diseased heart (*DSP* $^{p.Lys569X/WT}$) . Fibro-fatty and myocardial regions are depicted. Scale bars, 5 mm (top) and 100 µm (bottom). **(B)** Sanger sequencing traces of genomic DNA isolated from control and *DSP* $^{p.Lys569X/WT}$ heart. **(C)** Representative western blot of DSP (green arrows) showing the absence of a truncated variant (expected size is 65.7kDa, as indicated by the red arrow) in control and *DSP* $^{p.Lys569X/WT}$ LV tissue. **(D)** Quantification of DSP protein levels in control and *DSP* $^{p.Lys569X/WT}$ LV tissue. Vinculin (VIN) was used as loading control. DSPtv; DSP truncated variant.

Supplementary Figure 2

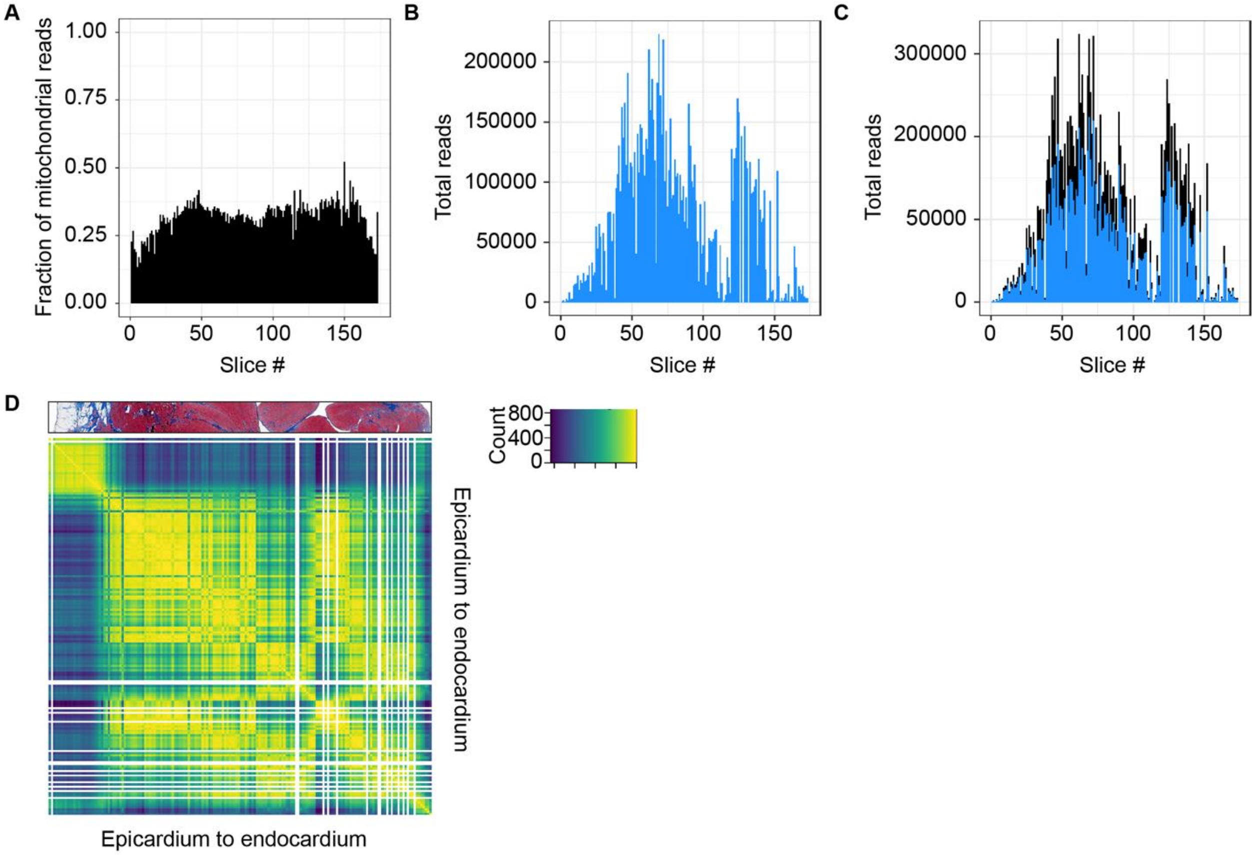

Figure S2. Spatial transcriptomics procedure and parameters to assess quality of data.

(A) Plot showing the fraction of mitochondrial genes per consecutive slices. **(B)** Plot showing the total amount of read counts, excluding mitochondrial reads per slice. **(C)** Plot showing the total amount of read counts excluding (in blue) and including (in black) mitochondrial reads. (**D)** Semi-supervised clustering of all sections that passed quality control. The physical order of the sections is retained. A representative histology image has been included for orientation.

Supplementary Figure 3

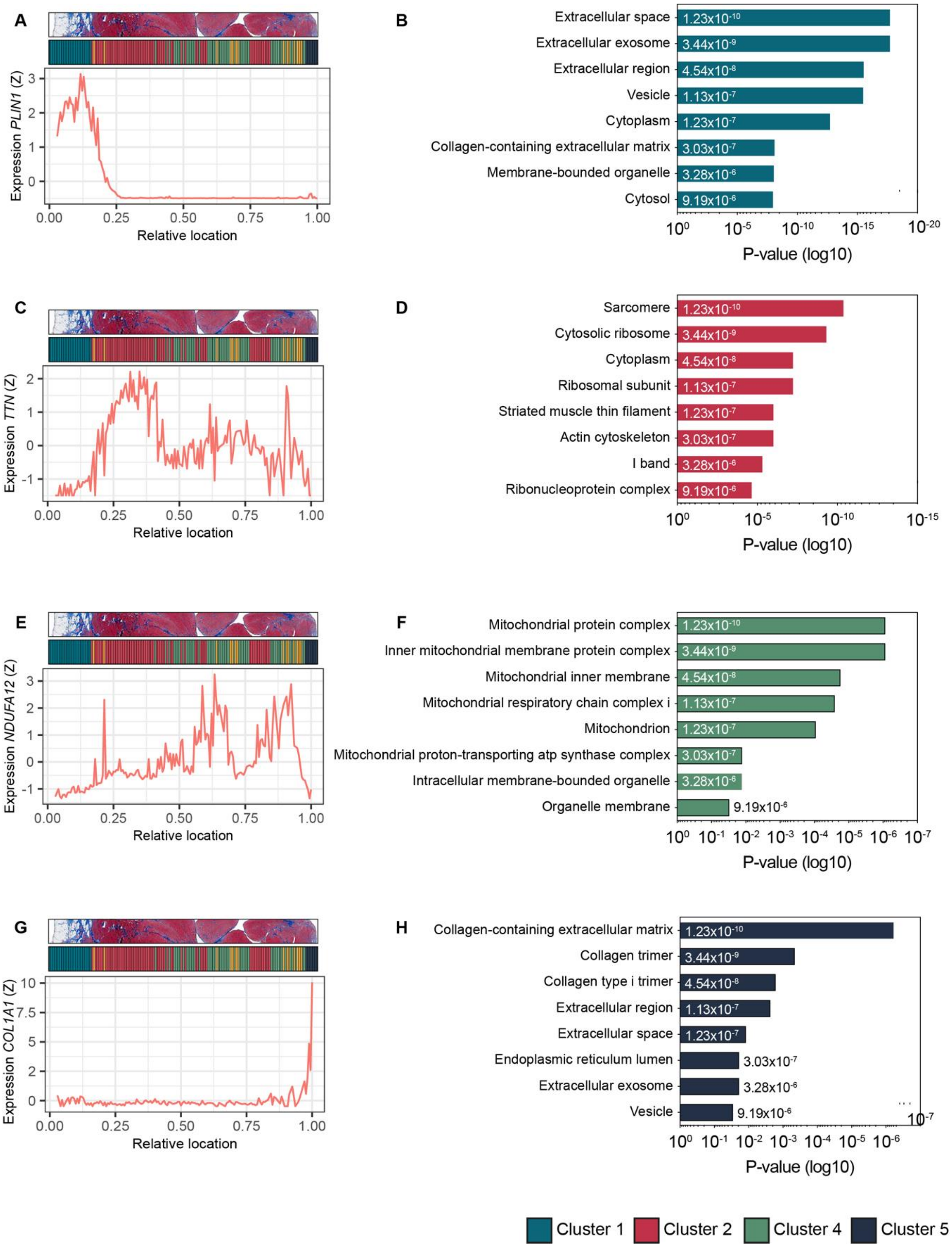

Figure S3. Functional annotation of the clusters identified in the *DSP* $^{p.Lys569X/WT}$ heart.

(A) Spatial expression pattern for perilipin-1 (*PLIN1*) across all sections showing enrichment in cluster 1. **(B)** Gene ontology analysis on genes upregulated in cluster 1 compared to all other clusters. **(C)** Spatial expression pattern for titin (*TTN*) across all sections showing enrichment in cluster 2. **(D)** Gene ontology analysis on genes upregulated in cluster 2 compared to all other clusters. **(E)** Spatial expression pattern for Endothelial PAS domain-containing protein 1 (*EPAS1*) across all sections showing enrichment in cluster 4. **(F)** Gene ontology analysis of genes upregulated in cluster 4 compared to all other clusters. **(G)** Spatial expression pattern for collagen alpha-1(I) chain (*COL1A1*) across all sections showing enrichment in cluster 5. **(H)** Gene ontology analysis on genes upregulated in cluster 5 compared to all other clusters. Genes were considered significant when log2FC > 1.00 and adjusted $p < 0.05$. For A, C and E, sections are ordered from the epi- (left) to endocardium (right), and a representative histology image has been included for orientation. Expression patterns were Z-score normalized. Cluster 3 did not show any enrichment with the parameters used.

Supplementary Figure 4

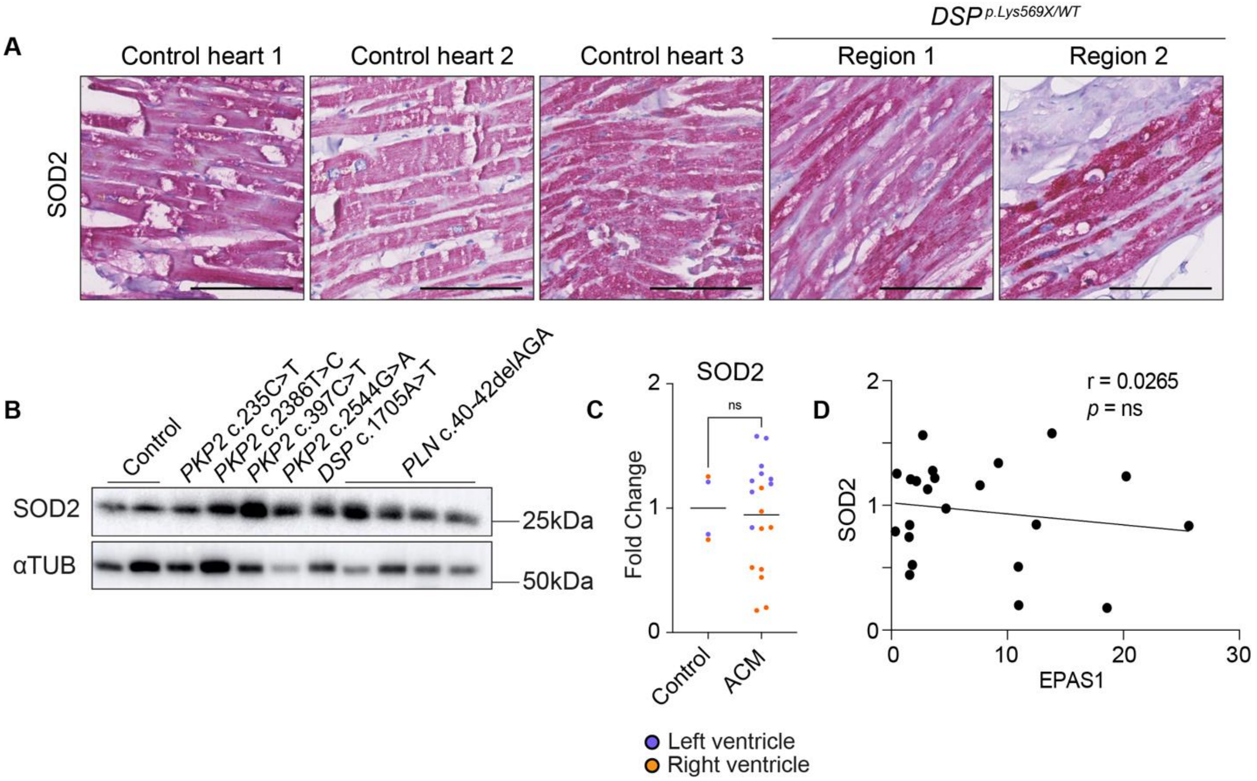

Figure S4. SOD2 is not induced in human explanted ventricular tissue obtained from patients diagnosed with either arrhythmogenic or dilated cardiomyopathy. **(A)** Immunohistochemistry for SOD2 on left ventricular tissue collected from explanted healthy and DSP p.Lys569X hearts. Scalebar: 100µm. **(B)** Representative western blot for SOD2 on left ventricular tissue obtained from explanted hearts with the indicated mutations. **(C)** Quantification of SOD2 protein levels in left- (purple dots) and right- (orange dots) ventricular tissue collected from healthy (two) individuals or patients diagnosed with arrhythmogenic (ACM) or dilated (DCM) cardiomyopathy bearing the indicated mutations. Alpha tubulin (αTUB) was used as a loading control. **(E)** Spearman's correlation analysis between EPAS1 and SOD2 protein levels of the subjects shown in (C). A two-tailed Mann-Whitney test was used to assess significance (**p*<0.05, ***p*<0.01).

Supplementary Figure 5

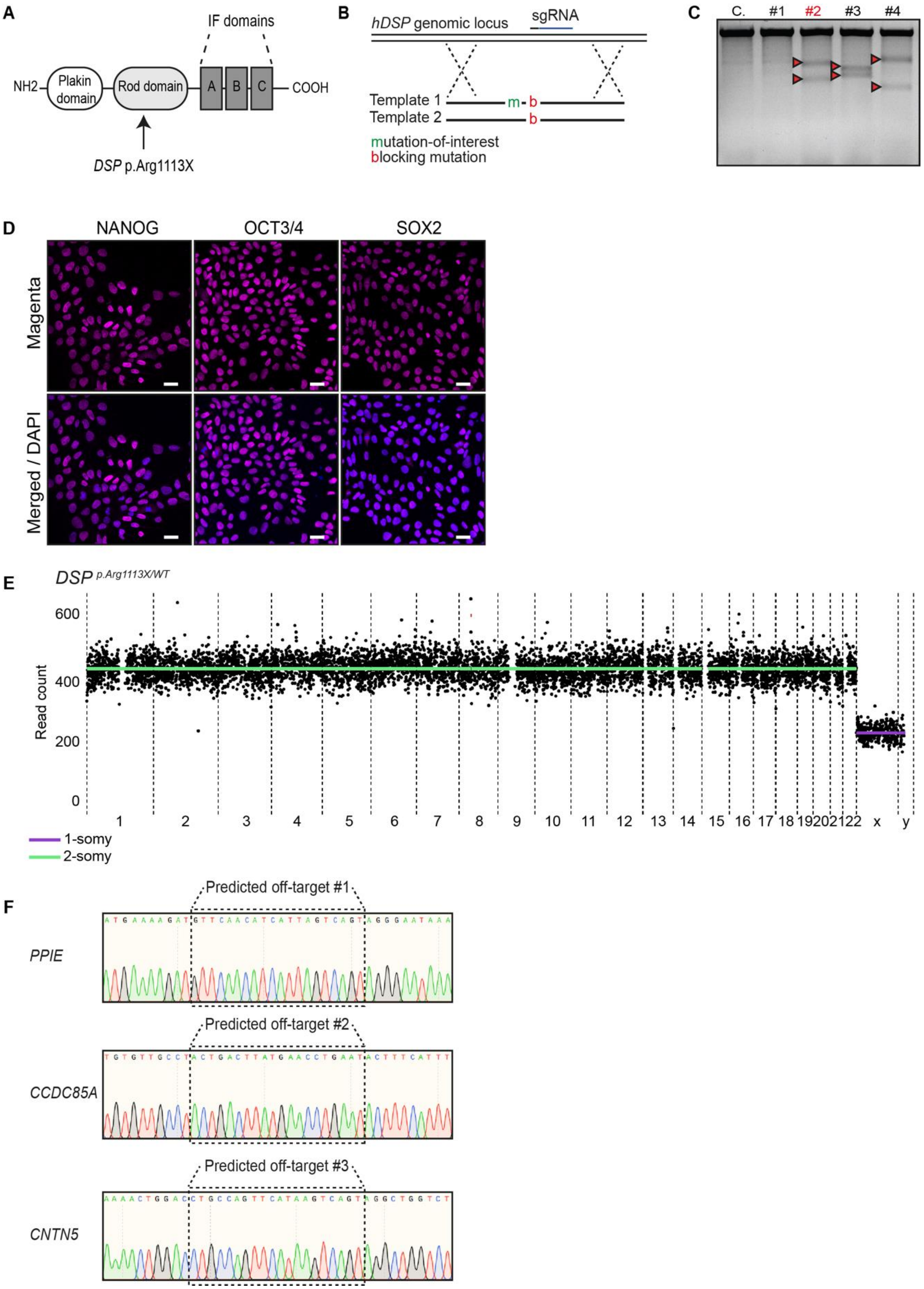

Figure S5. Characteristics of the *DSP* $^{p.Arg1113X/WT}$ knock-in hiPSC line.

(A) Scheme representing DSP. The mutation of interest is indicated with an arrow. IF domains; intermediate filament domains. **(B)** Scheme depicting the targeting strategy. **(C)** T7-endonuclease assay showing the cutting efficiency of four selected single guide RNAs. Single guide RNA #2 (in red) was selected for targeting healthy control hiPSCs. **(D)** Representative immunofluorescence pictures for NANOG, OCT3/4, SOX2 (in magenta) and DAPI (blue). Scalebar: 20 μm. **(E)** Plot showing the number of reads assigned to each chromosome (Karyo-sequencing profile). **(F)** Sanger traces for the predicted top three off-target loci. Dashed box indicates the potential binding site of single guide RNA #2 within *PPIE*, *CCDC85A* and *CNTN5*.

Supplementary Figure 6

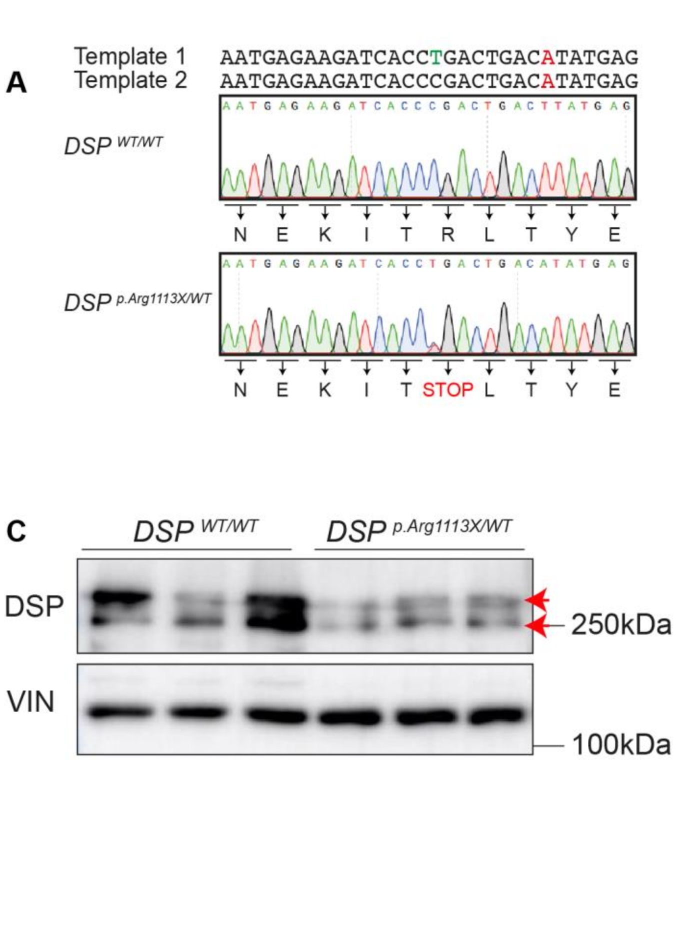

Figure S6. Heterozygous *DSP* $^{p.Arg1113X/WT}$ hiPSC-derived cardiomyocytes are DSP-haploinsufficient.

(A) Sequencing traces of the control and *DSP* $^{p.Arg1113X/WT}$ knock-in hiPSC lines. The DNA templates used to introduce the intended mutation are depicted. The intended and blocking mutation are indicated in green and red, respectively. **(B)** Gene expression levels for *DSP*. Values normalized to the housekeeping gene *GUS*. **(C)** Representative immunoblot for DSP (corresponding bands are indicated with red arrows) and the loading control VIN. **(D)** Quantification of DSP protein levels normalized to VIN. Colored dots represent different batches of differentiations (n=6 technical replicates and N=3 biological replicates). Data plotted as mean. A two-tailed unpaired Student's t-test or two-tailed Mann-Whitney test was used to assess significance (** *p*-value < 0.01, ns = non-significant).

Table S1. Clinical features of the patient bearing a *DSP* $^{p.Lys569X/WT}$ mutation.

Genetic mutation	*DSP c.1705A>T/WT*
Protein change	DSP p.Lys569X/WT
Age at transplant	44 years
Sex	Male
Cardiac abnormalities	- Microvoltages<br>- Delayed terminal activation<br>- Negative T-waves

DSP, desmoplakin; WT, wildtype.

Table S3. Single guide RNA sequences

Name	Sequence (5'->3')
DSP-KI_sgRNA1_FW	CACCgTCAATCTCATAAGTCAGT
DSP-KI_sgRNA1_RV	AAACACTGACTTATGAGATTGAC
DSP-KI_sgRNA2_FW	CACCgCTTCAATCTCATAAGTCAGT
DSP-KI_sgRNA2_RV	AAACACTGACTTATGAGATTGAAGC
DSP-KI_sgRNA3_FW	CACCgTCATTGAGTTCTTTTATT
DSP-KI_sgRNA3_RV	AAACAATAAAAGAACTCAATGAC
DSP-KI_sgRNA4_FW	CACCgCAATCTCATAAGTCAGTC
DSP-KI_sgRNA4_RV	AAACGACTGACTTATGAGATTGC

DSP; desmoplakin; FW, forward; sgRNA, single guide RNA; KI, knock-in; RV, reverse.

Table S4. Genotyping primers

Name	Sequence (5’->3’)
DSP-universal_FW	ATATGAGCAGCTGGTGCAAG
DSP-universal_RV	CTTGGCCTCCTCCTGAAAC

DSP; desmoplakin; FW, forward; RV, reverse.

Table S5. Single-stranded templates used for targeting

Name	Sequence (5'->3')
DSP_Arg1113X_ssODN1	GACAGGCTGAGCTGGATGGGAAGTCGGCTAAGCAAAATCTAGACAAGTGCTACGGCCAAATAAAAGAACTCAATGAGAAGATCACCTGACTGACATATGAGATTGAAGATGAAAAGAGAAGAAGAAA
DSP_Arg1113X_ssODN2	GACAGGCTGAGCTGGATGGGAAGTCGGCTAAGCAAAATCTAGACAAGTGCTACGGCCAAATAAAAGAACTCAATGAGAAGATCACCCGACTGACATATGAGATTGAAGATGAAAAGAGAAGAAGAAA

DSP; desmoplakin; ssODN, single-stranded oligodeoxynucleotide.

Table S6. Primers for amplification of off-target loci

Name	Sequence (5’->3’)
Off-target 1_FW	CACAGTGGCTGGACCATTTT
Off-target 1_RV	CCGTACCAATAGAGGGCAAA
Off-target 2_FW	CCGGGAATGAATTACAATGG
Off-target 2_ RV	CTACATCTCCGCCTCACCTC
Off-target 3_FW	GGCAGAGAACTGCTTGAACC
Off-target 3_RV	GAAATTGCTGACCCCTCTGA

FW, forward; RV, reverse.

Table S7. Quantitative PCR primers.

Name	Forward - sequence (5'->3')	Reverse - sequence (5'->3')
BCL2/Adenovirus E1B 19 kDa protein-interacting protein 3-like (BNIP3L)	TTGGATGCACAACATGAATCAGG	TCTTCTGACTGAGAGCTATGGTC
Natriuretic peptide A (NPPA)	CCGTGAGCTTCCTCCTTTTA	CCAAATGGTCCAGCAAATTC
Natriuretic peptide B (NPPB)	CTCCAGAGACATGGATCCCC	GTTGCGCTGCTCCTGTAAC
Growth arrest and DNA-damage-inducible alpha (GADD45α)	ACCATGCAGGAAGGAAAACT	TACCCAAACTATGGCTGCAC
Gamma-Aminobutyric Acid Receptor-Associated Protein Like 2 (GABARAPL2)	ATCTCCGAGCTGAGGATGCCTT	GACACTTTCGTCACTGTAGGCAA
Adrenomedullin (ADM)	ACGGCTTTGCACACGTAAAC	GCATCCGGACTGCTGTCTT
Microfibrillar-Associated Protein 5 (MFAP5)	TGGCTGATATTGCACCTTCCA	GGCCGATGCACAGAGTAGAG
Desmoplakin (DSP)	GCACCAGCAGGATGTACTATT	TCAATTCAGGCTGCACGAT
Endothelial PAS domain-containing protein 1 (EPAS1)	GGACTTACACAGGTGGAGCTA	TCTCACGAATCTCCTCATGGT
Glucuronidase Beta (GUS)	CCACCTAGAATCTGCTGGCTAC	GTGCCCGTAGTCGTGATACCAA
